# Network analysis of inflammation and symptoms in recent onset schizophrenia and the influence of minocycline during a clinical trial

**DOI:** 10.1038/s41398-023-02570-8

**Published:** 2023-09-18

**Authors:** Sarah E. Herniman, Stephen J. Wood, Golam Khandaker, Paola Dazzan, Carmine M. Pariante, Nicholas M. Barnes, Carl R. Krynicki, Naghmeh Nikkheslat, Rachel C. Vincent, Alex Roberts, Annalisa Giordano, Andrew Watson, John Suckling, Thomas R. E. Barnes, Nusrat Husain, Peter B. Jones, Eileen Joyce, Stephen M. Lawrie, Shôn Lewis, Bill Deakin, Rachel Upthegrove, the BeneMin Study Team

**Affiliations:** 1 Orygen, Melbourne, Australia; 2 Centre of Youth Mental Health, University of Melbourne, Melbourne, Australia; 3 Melbourne School of Psychological Sciences, University of Melbourne, Melbourne, Australia; 4 Institute for Mental Health and Centre for Human Brain Health, University of Birmingham, Birmingham, UK; 5 MRC Integrative Epidemiology Unit, Bristol Medical School, University of Bristol, Bristol, UK; 6 Centre for Academic Mental Health, Population Health Sciences, Bristol Medical School, University of Bristol, Bristol, UK; 7 Department of Psychiatry, University of Cambridge School of Clinical Medicine, Cambridge, UK; 8 Cambridgeshire and Peterborough NHS Foundation Trust, Cambridge, UK; 9 Avon and Wiltshire Mental Health Partnership NHS Trust, Bristol, UK; 10 Department of Psychological Medicine, Institute of Psychiatry, Psychology and Neuroscience, London, UK; 11 Stress, Psychiatry and Immunology Lab & Perinatal Psychiatry, The Maurice Wohl Clinical Neuroscience Institute, King’s College London, London, UK; 12 Institute of Clinical Sciences, College of Medical and Dental Sciences, University of Birmingham, Birmingham, UK; 13 The Department of Clinical and Motor Neurosciences, UCL Institute of Neurology, London, UK; 14 Brain Mapping Unit, Department of Psychiatry, Herchel Smith Building for Brain and Mind Sciences, Cambridge, UK; 15 Cambridgeshire & Peterborough NHS Foundation Trust, Cambridge, UK; 16 Imperial College London, Division of Psychiatry, London, UK; 17 Lancashire & South Cumbria NHS Foundation Trust, London, UK; 18 Division of Psychology and Mental Health, University of Manchester, Manchester, UK; 19 Division of Psychiatry, Centre for Clinical Brain Sciences, University of Edinburgh, Edinburgh, UK; 20 Early Interventions Service, Birmingham Womens and Children’s NHS Foundation Trust, Birmingham, UK

**Keywords:** Diagnostic markers, Biomarkers

## Abstract

Attempts to delineate an immune subtype of schizophrenia have not yet led to the clear identification of potential treatment targets. An unbiased informatic approach at the level of individual immune cytokines and symptoms may reveal organisational structures underlying heterogeneity in schizophrenia, and potential for future therapies. The aim was to determine the network and relative influence of pro- and anti-inflammatory cytokines on depressive, positive, and negative symptoms. We further aimed to determine the effect of exposure to minocycline or placebo for 6 months on cytokine-symptom network connectivity and structure. Network analysis was applied to baseline and 6-month data from the large multi-center BeneMin trial of minocycline (*N* = 207) in schizophrenia. Pro-inflammatory cytokines *IL-6, TNF-*α, and *IFN-γ* had the greatest influence in the inflammatory network and were associated with depressive symptoms and suspiciousness at baseline. At 6 months, the placebo group network connectivity was 57% stronger than the minocycline group, due to significantly greater influence of *TNF-α, early wakening*, and *pathological guilt*. *IL-6* and its downstream impact on *TNF-α*, and *IFN-γ*, could offer novel targets for treatment if offered at the relevant phenotypic profile including those with depression. Future targeted experimental studies of immune-based therapies are now needed.

## Introduction

Schizophrenia spectrum disorders (termed schizophrenia hereafter) are among the most burdensome psychiatric illnesses worldwide [1]. Despite the increased availability of antipsychotic medications, recovery rates have remained largely unchanged over the past four decades [2]. To improve outcomes in schizophrenia, continued research is needed to determine aetiological mechanisms and biomarkers, and guide targeted, adjunctive treatment.

Inflammation might contribute to the aetiology of schizophrenia [3–16]. Individuals have shown elevated levels of pro-inflammatory cytokines, and such elevations reduced in line with antipsychotic and antidepressant medications [4, 8, 17–19]. Pro-inflammatory cytokines have been associated with greater negative [9, 20–26], co-occurring depressive [12, 20] and overall symptoms [27]. Meta-analyses of the numerous, generally small-scale trials of various anti-inflammatory drugs and drugs with secondary anti-inflammatory effects have reported an overall potential benefit in schizophrenia [28–30]. Early studies with the anti-inflammatory antibiotic, minocycline, produced promising results and benefits have been reported in MDD [31]. However, the BeneMin trial, conducted across multiple sites in the UK, the largest and most robust study of adjunctive minocycline in schizophrenia, found no support for its efficacy in treating negative symptoms [32]. Almost none of the trials of anti-inflammatory agents in schizophrenia have attempted to select patients with evidence of current inflammation, and there is as yet no clinical profile robustly associated with inflammation.

Previous studies have primarily compared the mean levels of small and different sub-sets of individual cytokines in schizophrenia. However, different cytokines serve different biological functions, with some playing a more influential role in inflammation, by enhancing or suppressing the production of other cytokines (e.g., *IL-6*/*IL-6r (receptor)* often termed the “bandmaster” with functions including regulation of other cytokines, and both pro and anti-inflammatory actions [33]). Rather than just association with a single elevated cytokine (which might or might not be associated with other cytokines), schizophrenia presentation might be dependent upon an *influential* cytokine(s) responsible for activating a large network of cytokines and their secondary effects. Treatment targeted to influential cytokine(s) might represent an important inflammatory biomarker and a key target for immune-based therapies.

Most studies examining clinical associations of cytokines in schizophrenia have focused on total and subscale scores of symptom ratings rather than on *individual* symptoms. If inflammation is associated with a subset(s) of specific symptoms in schizophrenia, the use of sum scores in previous research may have obscured associations and reduced the power to detect them [34]. In addition, since inflammation occurs in MDD [8, 17, 34–42] and co-occurring depression in schizophrenia is common [43], previous associations between cytokines and negative [9, 20–26] and overall symptoms [27] in schizophrenia may have been influenced by unmeasured co-occurring depression [20]. Thus, research also needs to determine the associations between influential cytokine(s) and individual symptoms in schizophrenia, while controlling for the influence of each other.

Using data from the BeneMin trial, our primary aims were to determine: (1) the network and relative influence of a large set of both pro- and anti-inflammatory cytokines; and (2) the associations between influential cytokine(s) and individual symptoms, while controlling for the influence of other symptoms. As a secondary aim, while minocycline medication had no evidence of overall beneficial effect on symptoms, we also investigated whether it potentially weakened cytokine networks that relate to specific symptoms over 6 months [32].

## Methods

### Participants

This study involved analysis of baseline and 6-month data from our BeneMin Study [32, 44]. The eligibility criteria for BeneMin has been described in detail elsewhere [32]. In brief, participants were experiencing a recent-onset schizophrenia spectrum disorder including schizophrenia, schizophreniform, and schizoaffective disorder (<5 years) with active psychotic symptoms (defined ≥3 on positive symptom subscale [PANSS_P_] of the interviewer-rated Positive and Negative Syndrome Scale [45]: ‘delusions’, ‘conceptual disorganisation’, ‘hallucinatory behaviour’, or ‘suspiciousness/persecution’). Diagnoses were based on Diagnostic and Statistical Manual of Mental Disorders—Fourth Edition [DSM-IV; 48] and confirmed using the Mini-International Neuropsychiatric Interview [MINI; [46]].

For the current purposes, participants with baseline CRP ≥ 10 mg/L were excluded to avoid potential biases related to acute inflammatory infection [34].

### Procedure

All participants provided oral and written informed consent and all procedures were conducted in accordance with protocols approved by The North West Manchester Research Ethics Committee [refer to [32, 44]].

### Measures

#### Inflammatory cytokines

Plasma samples were collected, prepared, and frozen. Cytokines were analysed using Meso Scale Discovery sandwich immunoassays. V-PLEX Plus Proinflammatory Panel 1 Human Kit was used to detect the levels of the following pro-inflammatory cytokines *hsCRP*, IL-1beta [*IL-1β*], *IL-2, IL-6, IL-8, IL-12, TNF-α*, interferon-gamma [*IFN-γ*], and anti-inflammatory cytokines including *IL-4* and *IL-10*. Plates read on an MSD QuickPlex SQ 120, as previously conducted [47, 48]. The results were analysed using MSD DISCOVERY WORKBENCH analysis software. *IL-1RA* were measured using R&D ELISA kit. Assays were performed at King’s College London.

#### Symptomatology

##### Positive and Negative Syndrome Scale

The PANSS_P_ and PANSS negative symptom subscale (PANSS_N_) were used to measure positive and negative symptoms. The PANSS_P_ and PANSS_N_ comprise 7 items rated on a 7-point Likert scale, ranging from 1 (absent) to 7 (extremely severe). PANSS_N_ items include: ‘blunted affect’, ‘emotional withdrawal’, ‘poor rapport’, ‘passive/apathetic social withdrawal’, ‘difficulty in abstract thinking’, ‘lack of spontaneity and flow of conversation’, and ‘stereotyped thinking’. PANSS_P_ items include: ‘delusions’, ‘conceptual disorganisation’, ‘hallucinatory behaviour’, ‘excitement’, ‘grandiosity’, ‘suspiciousness/persecution’, and ‘hostility’ [49]. Scores on both the PANSS_P_ and PANSS_N_ range from 1 to 49, with higher scores indicating more severe symptomatology.

##### Calgary Depression Scale for Schizophrenia

The Calgary Depression Scale for Schizophrenia [CDSS; [50]] was used to measure depressive symptoms. The CDSS has the greatest psychometric properties to measure depressive symptoms in schizophrenia [51], and is used most frequently for such purposes [43]. It was therefore used in the current study instead of depression items on the PANSS. It is an interviewer-rated scale comprising 9 items rated on a 4-point Likert response scale, ranging from 0 (absent) to 3 (severe). Items include: ‘depression’, ‘hopelessness’, ‘self-deprecation’, ‘guilty ideas of reference’, ‘pathological guilt’, ‘morning depression’, ‘early wakening’, ‘suicide’, and ‘observed depression’. Scores range from 0 to 27, with higher scores indicating more severe symptomatology.

#### Covariates

In addition to controlling for the influence of all other symptoms, age, sex, and body mass index (BMI) were also entered as covariates in the networks, described below.

### Statistical analyses

All statistical analyses were conducted in *R* (Version 4.0.3). Data and assumption screening were undertaken and descriptive statistics and frequency counts were obtained for demographic and clinical characterization [52, 53].

#### Network estimation and accuracy and stability testing

In accordance with recommendations [54, 55], regularized, partial association (here, Spearman’s correlation [*r*] was used because of positively skewed variables) networks using the Least Absolute Shrinkage and Selection Operator [LASSO; [56]] in combination with the Extended Bayesian Information Criterion [EBIC [57]] were estimated. While *r* ranges between −1.00 and 1.00, the EBIC LASSO procedure maximizes specificity of such associations or edge weights (i.e., aims to include as few *false positives* as possible) by shrinking them towards zero and setting trivially small and therefore spurious associations to exactly zero. This results in a sparse graphical model comprising only the strongest associations. Given this, the strength of non-zero, regularized partial associations (*r* hereafter) are not equivalent to Cohen’s criteria. Rather, all *r* are considered sufficiently strong and meaningful [54, 55]. The EBIC tuning hyperparameter λ was set to 0 to maximize discovery of such non-zero, regularized associations (rather than λ = 0, which maximizes caution, even after regularization).

The networks presented illustrate *r* among variables, and this is depicted by nodes (circles representing cytokines or symptoms) and edges (lines representing *r*). Thicker edges denote stronger associations among cytokines or symptoms. To plot networks, the circle layout was used for ease of interpretation and to allow for comparison across networks.

The accuracy and stability of the resulting edge weights were examined using nonparametric and case-dropping subset bootstrapping procedures [54, 55]. In relation to nonparametric procedures, we specifically report how often each edge-weight of interest (e.g., *r* between an influential cytokine(s) and symptom) was estimated as >0 in the 1000 nonparametric bootstrapped samples. Edge weights that are present in at least 50% of the 1000 nonparametric bootstrap samples are considered reliably present, and vice-versa. In relation to case-dropping procedures, the correlation stability co-coefficient (CS-coefficient) was calculated, which refers to the proportion of cases that can be dropped while still maintaining (with 95% certainty) a correlation of at least 0.70 with observed network coefficients. Here, the CS-coefficient should not be below 0.25, and ideally above 0.5 [54, 55].

This approach to network estimation, accuracy, and stability testing was applied across all four networks, discussed in detail below (Table 1). The first and second networks (Network 1 & 2) correspond to the primary aims of the current study, and were derived from baseline data. Networks 3 and 4 correspond to the secondary aims, and were derived from 6-month data.

**Table 1:** Overview of network models.

Network	Description	Sample	Variables
*Baseline*			
1	Cytokine network and relative influence of cytokines	All, *N* = 194	All cytokines
2	Influential cytokines and associations with subgroups of specific symptoms	All	Influential cytokine(s); all symptoms; covariates
*Six-month follow-up*			
3	Treatment specific effects on the cytokine-symptom network	All	Treatment allocation (placebo/minocycline); influential cytokine(s); relevant symptom(s); covariates
4	Differences in the connectivity of the cytokine-symptom network between treatment groups	Placebo versus minocycline	Influential cytokine(s); relevant symptom(s); covariates

##### Network 1: Cytokine network and relative influence of cytokines at baseline

We first estimated the cytokine network and relative influence of a large set of pro-inflammatory cytokines, including *hsCRP, IL-1β*, *IL-2, IL-6, IL-8, IL-12, IL-13, TNF-α, IFN-γ* and anti-inflammatory cytokines, including *IL-1RA*, *IL-4, IL-10*. To determine the relative influence of each, strength centrality was calculated, which is the sum of the strength of associations between a cytokine and all other cytokines to which it is associated [54, 55]. Here, we follow recent recommendations and convention in network analysis, selecting and interpreting the top scoring cytokines as most meaningful, based on z-transformed, distributional tendencies in combination with disciplinary expertise [58, 59].

##### Network 2: Influential cytokines and associations with subgroups of specific symptoms at baseline

Network 2 estimated associations between influential cytokine(s), derived by Network 1, and subgroups of specific symptoms (items CDSS, PANSS_P_, PANSS_N_) and covariates (age, BMI, sex) at baseline. To determine subgroups of symptoms, community analysis (an empirically driven technique to determine clusters of symptoms) was conducted using the well-established spinglass algorithm [60]. The algorithm was conducted 1000 times, and the median number of communities was obtained. A seed was then found that produced this median number of communities, and consequently set to allow for replicability [60]. Here, we explicitly report all non-zero *r* between cytokines and subgroups of specific symptoms.

**Table 2:** Baseline demographic and clinical characteristics and inflammatory biomarkers (*N* = 194).

Variable	*M* (*SD*)
*Demographic characteristics*	
Age	25.58 (5.19)
Sex (female)	25.8% (*n* = 50)
BMI	27.35 (6.53)
*Inflammatory biomarkers*	
hsCRP (mg/L)	2.52 (2.35)
IL-1RA	407.49 (437.60)
IL-1β (pg/L)	0.07 (0.26)
IL-2	0.23 (0.26)
IL-4	0.02 (0.02)
IL-6	0.70 (0.50)
IL-8	4.51 (2.95)
IL-10	0.44 (0.89)
IL-12	0.14 (0.16)
IL-13	0.49 (0.48)
TNF-α	2.50 (0.64)
IFN-γ	4.80 (5.93)
*Clinical characteristics*	
PANSS_P_ total score	16.79 (4.72)
Placebo group (*n* = 95)	17.22 (5.29)
Minocycline group (*n* = 99)	16.37 (4.09)
PANSS_N_ total score	17.25 (5.71)
Placebo group	16.76 (5.42)
Minocycline group	17.72 (5.97)
CDSS total score	5.36 (4.62)
Placebo group	5.44 (4.99)
Minocycline group	5.21 (4.99)
GAF total score	56.03 (10.10)
Placebo group	56.86 (10.94)
Minocycline group	55.34 (9.23)

*BMI* body mass index, *hsCRP* high sensitivity C-reactive protein, *IL* interleukin, *IL-1RA* interleukin-receptor antagonist, *IL-1β* interleukin-beta, *TNF-α* tumor necrosis factor alpha, *IFN-γ* interferon gramma, *PANSS* Positive and Negative Syndrome Scale, *PANSS*_*N*_ negative subscale of the PANSS, *PANSS*_*P*_ positive subscale of the PANSS, *CDSS* Calgary Depression Scale for Schizophrenia, *GAF* Global Assessment of Functioning.

##### Network 3: Treatment effects on the cytokine-symptom network at 6-month follow-up

At 6-month follow-up, to identify its action, minocycline was added as a node (placebo[1]/minocycline[2]) in a network of influential cytokines and relevant symptoms while controlling for covariates. Only relevant symptoms were included due to sample size limitations that precluded simultaneous network estimation of all symptoms by treatment subgroups [54, 55]. Relevant symptoms were defined as those belonging to a community that was associated with an influential cytokine(s), as derived in Network 2. As done in previous studies [61], we controlled for baseline overall symptom severity by including PANSS total score as a node in the network. Comparison between treatment groups with different overall severity or differences in unassessed latent constructs might result in different connectivity between symptoms solely due to differences in variances [62]. Here, a direct association between treatment and a cytokine or symptom indicates a direct cytokine- or symptom-specific effect of treatment, independent of the effects on other cytokines or symptoms [63]. If treatment has a direct cytokine- but not symptom-specific effect, and cytokines have a direct symptom-specific effect, it can be interpreted that treatment is associated with symptoms of schizophrenia *via* cytokines [63].

##### Network 4: Differences in the connectivity and structure of the cytokine-symptom network between treatment groups at 6-month follow-up

Finally, the Network Comparison Test (NCT [61]) was conducted to compare the connectivity (or global strength, the sum of all absolute edge weights) and structure (or network invariance, distributions of edge weights) of cytokine-symptom networks between treatment groups (placebo versus minocycline) while controlling for covariates. We first tested the necessary (split-half) reliability assumption that networks were similar in terms of global strength and network invariance between groups at baseline, before testing this within groups (baseline versus 6-month follow-up) and between groups at 6-month follow-up. The NCT is a two-tailed permutation test, where differences between treatment groups were calculated 1000 times for randomly regrouped individuals. The resulting distribution was then used to test the observed differences between treatment groups [61]. The test of global strength produces the *S* statistic, denoting the difference in global strength between networks. The network invariance test produces the *M* statistic, which is the maximum difference in any of the edge weights. In the case of statistical significance, tests for differences in strength centrality and edge invariance are, respectively, recommended [61].

## Results

### Participant flow

Of the 207 BeneMin participants, 6.3% (*n* = 13) were excluded at baseline due to CRP ≥ 10 mg/L. The final baseline sample comprised 194 individuals with recent-onset schizophrenia and CRP < 10 mg/L. Excluded participants did not differ from the analytic sample in terms of depressive (respectively, CDSS *Mean* = 6.46 & *M* = 5.36; *t*(13.32) = −0.75, *p* = 0.466), positive (PANSS_P_ *M* = 17.46 & =16.79; *t*(13.33) = −0.45, *p* = 0.661), or negative symptoms (PANSS_N_ *M* = 17.08 & *M* = 17.25; *t*(13.84) = 0.11, *p* = 0.915).

### Sample characteristics

Table 2 presents demographic and clinical characteristics of the current cohort. The average age of participants was 25.58 years (*SD* = 5.19) and most were male (74.2%, *n* = 144).

### Networks

#### Network 1: Cytokine network and relative influence of cytokines at baseline

**Fig. 1:**
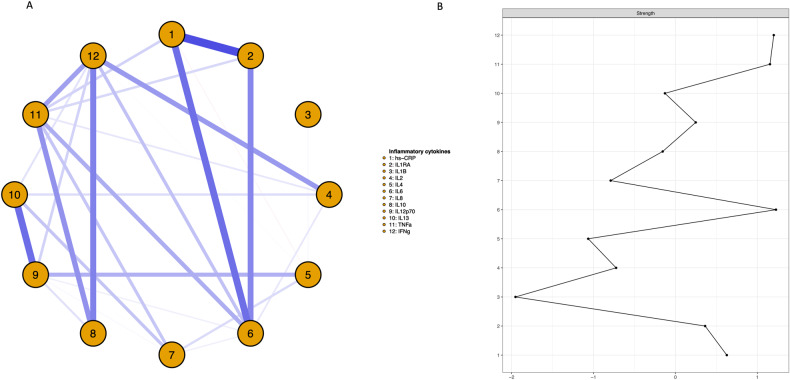
Cytokine network and relative influence of cytokines at baseline. **A** Baseline network displaying associations among pro- and anti-inflammatory cytokines in recent-onset schizophrenia spectrum disorders. Blue edges represent positive partial correlations (conditional dependence associations), and red edges represent negative partial correlations. Thicker edges represent stronger correlations. **B** The strength centrality indices (*z*-scores) for each cytokine.

Figure 1 presents the network of pro- (*hsCRP*, *IL-1β*, *IL-2, IL-6, IL-8, IL-12, IL-13, TNF-α, IFN-γ*) and anti-inflammatory cytokines (*IL-1RA*, *IL-4, IL-10*; Fig. 1A, left panel) as well as strength centrality (z-scores) for each cytokine (Fig. 1B, right panel). In Fig. 1A, blue edges represent positive *r* and red edges negative *r*, with thicker edges representing stronger associations. *IL-6, IFN-γ* and *TNF-α* had the greatest strength centrality of 1.23, 1.2, and 1.15, respectively. Strength centrality for all other cytokines was substantially lower, <0.63. This indicates that the influence of *IL-6, IFN-γ*, and *TNF-α* in the inflammatory network is ≥1.15 standard deviations than the average of all other pro- and anti-inflammatory cytokines in schizophrenia, and is consistent with previous research indicating that such cytokines have a central role in inflammation in schizophrenia [64–66]. *IL-6, IFN-γ* and *TNF-α* were associated with more than half of all other cytokines in the network (63.6%, 7 of a possible 11 associations). Thus, *IL-6, IFN-γ* and *TNF-α* were interpreted as the most influential cytokines, and included in remaining analyses.

#### Network 2: Influential cytokines and associations with subgroups of specific symptoms at baseline

**Fig. 2:**
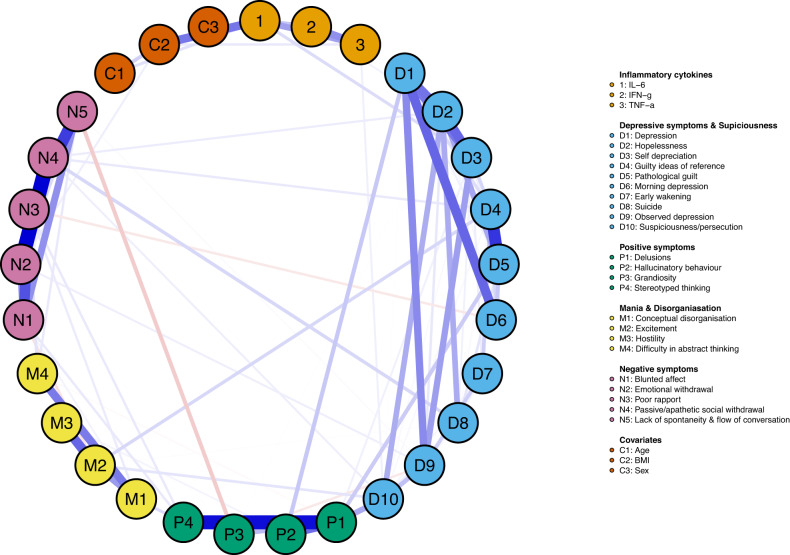
Influential cytokines and associations with subgroups of specific symptoms at baseline. Blue edges represent positive partial correlations (conditional dependence associations), and red edges represent negative partial correlations. Thicker edges represent greater correlations. Age and BMI were identified as belonging to the pro-inflammatory cytokines community, and sex as belonging to its own. However, for ease of visual interpretation, all covariates were plotted to comprise their own community—sitting next to pro-inflammatory cytokines—in the network.

Figure 2 presents the network of associations among influential cytokines and items of the CDSS, PANSS_P_ and PANSS_N_ (panel A, left) while controlling for covariates (age, BMI, sex). Six subgroups of cytokines or symptoms were identified and interpreted as: (1) *pro-inflammatory cytokines*; (2) *depression and suspiciousness*; (3) *positive symptoms*; (4) *mania and disorganisation*; (5) *negative symptoms*; and (6) *sex*. Age and BMI were identified as belonging to the pro-inflammatory cytokines community, and sex as belonging to its own. However, for ease of visual interpretation, all covariates were plotted to comprise their own community sitting next to pro-inflammatory cytokines (this does not impact associations between cytokines and symptoms).

*IL-6* was associated with *self-deprecation* (*r* = 0.06), *IFN-γ* with *hopelessness* (*r* = 0.02) and *TNF-α* with *suspiciousness* (*r* = 0.03). All three non-zero, cytokine-symptom associations were reliable. The *IL-6—self-depreciation* edge-weight was estimated to be above zero in 81% of the 1000 nonparametric bootstrapped procedures, *IFN-γ—hopelessness* in 64%, and *TNF-α—suspiciousness* in 63%. No other cytokine-symptom associations emerged (all *r* = 0.00). *Self-deprecation, hopelessness* and *suspiciousness* together with other CDSS items formed a distinct community, hereafter termed ‘depression and suspiciousness’. Thus, in addition to influential cytokines, symptoms of the depression and suspiciousness community were included in the networks at 6-month follow-up.

#### Network 3: Treatment specific effects on the cytokine-symptom network at 6-month follow-up

**Fig. 3:**
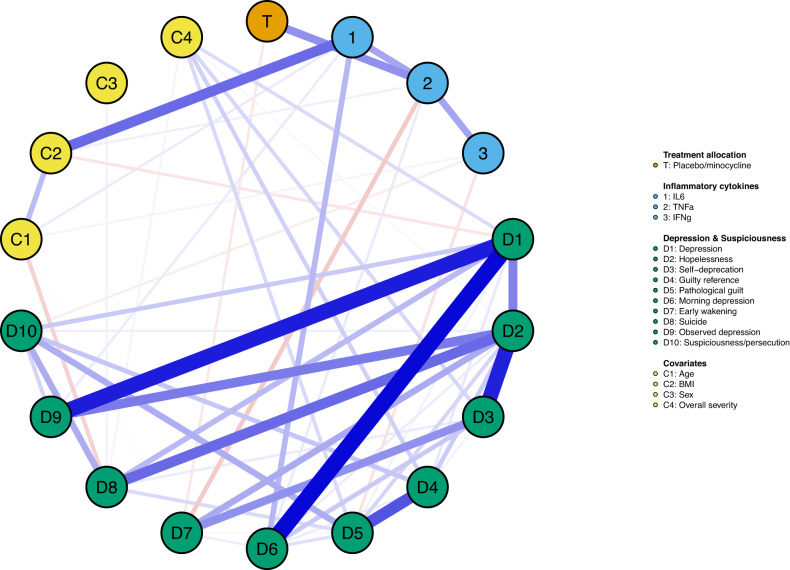
Treatment effects on the cytokine—symptom network at six-month follow-up. Blue edges represent positive partial correlations, and red edges represent negative partial correlations. Thicker edges represent greater correlations.

As seen in Fig. 3, minocycline interacted with the network exclusively through a positive association with *TNF-α* (*r* = 0.17) and negative association with *early wakening* (*r* = −0.04). These associations were reliable. The *treatment–TNF-α* association was estimated to be above zero in 96% of the 1000 nonparametric bootstrapped procedures, and *treatment–early wakening* in 60%. No other direct *treatment–cytokine* or *treatment-symptom* associations emerged (all *r* = 0.00).

#### Network 4: Differences in the connectivity and structure of the cytokine-symptom network between treatment groups

**Fig. 4:**
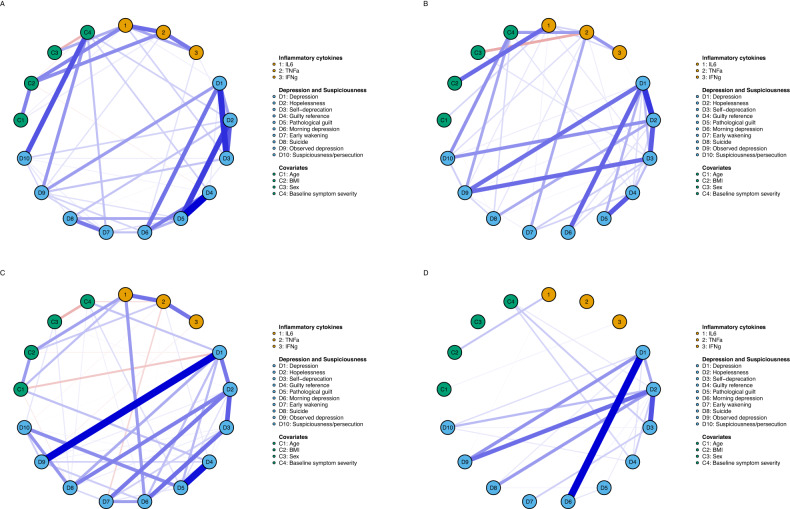
Differences in the connectivity and structure of the cytokine—symptom network between treatment groups at 6-month follow-up. Network of influential pro-inflammatory cytokines and symptoms of the depression and suspiciousness community at baseline and 6 months (baseline, top rows: **A** and **B**; 6 months, bottom rows: **C** and **D**) in the placebo (left columns, **A** and **C**) and minocycline (right column, **B** and **D**) groups. Blue edges represent positive partial correlations, and red edges represent negative partial correlations. Thicker edges represent stronger associations.

Figure 4 presents the network of influential pro-inflammatory cytokines and symptoms of the depression and suspiciousness community at baseline and 6 months (baseline, top rows: Fig. 4A, B; 6 months, bottom rows: Fig. 4C, D) in the placebo (left column, Fig. 4A, C) and minocycline (right column, Fig. 4B, D) groups. At baseline, there was no significant differences between the networks, in terms of global strength (placebo = 5.79, minocycline = 4.48), *S* = 1.31, *p* = 0.329, and structure, *M* = 0.31, *p* = 0.139. This indicates that networks were similar between groups at baseline, indicating split-half reliability. Given this, we did not test for specific differences in strength centrality or edge weights [61].

In the placebo group (Fig. 4A, C), the cytokine-symptom network at baseline appeared similar to the 6-month network, with the exception of greater strength of associations among cytokines and symptoms (depicted by thicker edges, Fig. 4C). *TNF-α* remained connected to symptoms and other influential cytokines, and this finding was reliable; the *TNF-α–IL-6* association was estimated to be above zero in 98% of the 1000 nonparametric bootstrapped procedures, and *TNF-α–IFN-γ* in 97%. From baseline to 6 months, the global strength of the network increased by 6.3% (5.79 to 6.16), *S* = 0.37; however, this within-group difference was statistically non-significant, *p* = 0.750. There were no significant differences in network invariance between baseline and 6-month follow-up, *M* = 0.33, *p* = 0.217.

In the minocycline group (Fig. 4B, D) at 6 months, there was a loss of connectivity between the three cytokines; *TNF-α* as well as *IFN-γ* were completely disconnected from the network and one another (all *r* = 0.00, Fig. 4D). These disconnections were reliable; the *TNF-α–IL-6* association was estimated to be above zero in less than 50% (49%) of the 1000 nonparametric bootstrapped procedures, *TNF-α–IFN-γ* in 28%, and *IFN-γ–IL-6* in 41%. Only one cytokine-symptom connection remained, *IL-6—observed depression* (*r* = 0.01); and this was reliable (appearing in 56% of bootstrapped procedures). From baseline to 6 months, the global strength of the network decreased by 61.0% (4.48 to 2.76), *S* = 1.73; however, this was statistically non-significant, *p* = 0.265. There were no significant differences in network structure between baseline and 6-month follow-up, *M* = 0.26, *p* = 0.432.

When directly comparing treatment groups at 6-month follow-up, the networks were significantly different from one another in terms of connectivity (57.6% greater in the placebo [6.16] compared to minocycline group [2.76]), *S* = 3.41, *p* = 0.049, and structure, *M* = −0.44, *p* = 0.032. Given this, we tested for differences in strength centrality and edge invariance [61]. Strength centrality of *TNF-α, early wakening*, and *pathological guilt* was significantly greater in the placebo (0.26, −0.08, 0.74) than minocycline group (−0.86, −0.62, −0.58; *p* < 0.001, *p* = 0.044, *p* = 0.026, respectively). *TNF-α* had a significantly stronger association with *IL-6* in the placebo (*r* = 0.26) than minocycline group (*r* = 0.00), *p* = 0.014. *Morning depression* had a significantly greater association with *early wakening* (placebo *r* = 0.18 versus minocycline *r* = 0.00, *p* = 0.029) and *self-depreciation* (placebo *r* = 0.27 versus minocycline *r* = 0.00, *p* = 0.012), and *pathological guilt* with *guilty reference* (placebo *r* = 0.44 versus minocycline *r* = 0.00, *p* = 0.014).

As a comparison, we also completed between group analyses for networks comprising our identified influential pro-inflammatory cytokines and positive symptoms. Details and results are reported in Supplementary. In summary, there was no disconnection of networks in either minocycline or placebo groups.

### Accuracy and stability analyses

In addition to the reliable edge weights of interest (reported above), accuracy and stability analyses also revealed adequate CS-coefficients, ranging between 0.36 and 0.67 (See Supplementary).

## Discussion

### Cytokine network in schizophrenia, and associations with subgroups of specific symptoms (baseline)

We found that pro-inflammatory cytokines *IL-6, TNF-*α and *IFN-γ* had the greatest influence (relative to other cytokines) in a network involving a large set of both pro- and anti-cytokines in schizophrenia. *IL-6, TNF-*α and *IFN-γ* were associated with depression and suspiciousness, but not to other positive or negative symptoms, while controlling for all other symptoms in the network.

The finding that pro-inflammatory cytokines *IL-6, TNF-*α and *IFN-γ* had the greatest influence in the network is consistent with previous research that has demonstrated elevated mean levels of pro-inflammatory cytokines in individuals with schizophrenia [3–16, 19]. The current findings also corroborate basic mechanistic work, indicating that *IL-6* is a central activator in the immune pathway, with previous research suggesting a causal pathway with schizophrenia and depression; in mendelian randomization studies, Khandaker et al. report that the functional variant of *IL-6R* gene is associated with decreased risk of severe depression and psychosis [66]. Two other Mendelian randomisation studies also report potentially causal role for *IL-6* in schizophrenia [64, 65]. Results in the present study reflect this evidence, since *IL-6* exerts a downstream effect on *TNF-*α and *IFN-γ*, therefore playing an *influential* role in a non-resolving pro-inflammatory state in schizophrenia [67]. This adds further to the suggestion that targeting *IL-6* may weaken a pro-inflammatory state and influential pro-inflammatory cytokines *IL-6, TNF-*α and *IFN-γ* might represent important biomarkers, and key targets for immune-based therapies in schizophrenia.

The finding that influential pro-inflammatory cytokines *IL-6, TNF-*α, or *IFN-γ* were associated with symptoms of depression (*self-depreciation* and *hopelessness*) and suspiciousness, but not to hallucinations, delusions, or negative symptoms, is also consistent with finding that inflammatory markers were associated with depressive symptoms in schizophrenia [12, 20]. However, previous studies also report that cytokines were associated with negative [9, 20–26] and overall symptoms [27]. These inconsistent findings could be attributable to methodological differences between the current multivariate analysis and previous smaller studies. When examining cytokine-symptom associations, previous studies also did not control for the influence of co-occurring depressive symptoms, or a number of confounds, whereas the current study did. It is therefore possible that previous associations were indicative of *secondary* negative symptoms (i.e., secondary to depression) or severity of overall symptoms [20]. Indeed, results in this study suggest that pro-inflammatory cytokines were directly associated with depression and suspiciousness, and, in turn, with positive and negative symptoms in schizophrenia.

These findings provide some support for a model of affective dysfunction in early stages of non-affective psychoses, such as schizophrenia [68]. The affective pathway to non-affective psychosis hypothesises that stressful life events can lead to psychotic symptoms *via* affective disturbances including depression. The current findings also extend such hypotheses, by suggesting that inflammation might be involved in this process. Inflammation has been associated with increased risk for MDD [69], and MDD with increased risk for transition to psychotic disorder [70, 71]. Thus, the presence of co-occurring depression might represent an inflammatory endophenotype of schizophrenia.

Given that influential pro-inflammatory cytokines *IL-6, TNF-*α and *IFN-γ* might represent important inflammatory biomarkers, and co-occurring depression might represent an inflammatory endophenotype of schizophrenia, immune-based therapies could target such biomedical profile in precision medicine studies. It would be expected that immunotherapeutic treatment would result in weaker connectedness or disintegration of the inflammatory–depressive-symptom network over time. Our secondary analysis of the BeneMin study tested this hypothesis, providing initial evidence of the potential for such an approach: we examined the effects of minocycline on the inflammatory–depressive network, and differences in network connectivity and structure between treatment groups (minocycline versus placebo) at 6-month follow-up [61].

### Effects of minocycline on the inflammatory–depressive-symptom network (6-month follow-up)

In contrast to the placebo group, in the minocycline group *IL-6, TNF-α* and *INF-γ* completely disconnected from one another, and from nearly all depressive symptoms at 6-month follow-up (Fig. 4D). Figure 3 suggests that minocycline impacted *TNF-*α and *early wakening* on the network shown in Figs. 3 and 4C to produce such disconnection in Fig. 4D. Several studies suggest that minocycline acts to reduce *TNF-α* expression as part of its anti-inflammatory actions [72]. In our primary BeneMin trial [32], minocycline had no overall effect on levels of *TNF-*α or other cytokines or symptoms, nevertheless, the present results suggest that minocycline may modify cytokine function and sleep, and through this its interaction with other symptoms.

Differences in the connectivity of the inflammatory–depressive symptom network were not statistically significant within groups over time but were statistically significant between groups at 6-month follow-up. In the placebo group, connectedness of the network *increased* by 6.3% over time, whereas connectedness of the network *decreased* by 61% in the minocycline group. At 6 months, network connectivity was significantly greater by 57.6% in the placebo compared to minocycline group, due to significantly greater influence of *TNF-α, early wakening* and *pathological guilt*. While there were no significant differences in the mean levels of cytokines or symptoms between groups at 6 months [32], these connectivity differences are equivalent to those seen in individuals with chronic versus remitted MDD [61] and could contribute to the antidepressant effects of minocycline reported in some MDD studies [73].

Two meta-analyses in schizophrenia [74, 75] reported that minocycline was associated with a reduction, albeit a statistically non-significant reduction, in levels of co-occurring depressive symptoms. Thus, findings together indicate that minocycline might exert effects onto the inflammatory–depressive-symptom network in schizophrenia, helping to significantly weaken such a network, but that such weakening does not necessarily translate into significant improvement in overall depressive symptoms at the group level. There are two potential explanations for this. First, since mechanisms other than inflammation might contribute to depression, improvements in symptoms might only be detectable in subgroups of individuals selected for immune active symptoms and evidence of immune activation. Indeed, a recent randomized controlled trials (RCT) of minocycline in MDD found significant improvement in symptoms in only those with immune activation (CRP ≥ 3 mg/L[33]). Second, while minocycline might weaken a depression symptom network, the finding that *IL-6* remained connected to the network indicates that minocycline might not supress all immune activation. Since *IL-6* might exert downstream effects onto *TNF-*α and *IFN-γ*, immune-based therapies targeting *IL-6* might be most beneficial. Furthermore, depressive symptoms remained connected to one another and this may indicate that even if there was complete severance of inflammatory contributions, depressive symptoms might nonetheless continue to maintain one another [76–79]. Minocycline could be most beneficial in individuals with depression and immune activation in the very early stages of illness, at a time before the chronicity of a self-maintaining symptom network is ‘set’. Indeed, Betz et al. demonstrate recent evidence consistent with this in a longitudinal network analysis of symptoms in clinical high risk and very recent-onset psychosis, highlighting the importance of depression in early stages in path to positive and negative symptom network strength over time [80].

### Limitations

The current study has some important limitations. First, participants with CRP levels ≥10 mg/L were excluded to avoid bias due to potential acute infection. Such a cut-off does not necessarily exclude all individuals with minor acute illnesses, which could have influenced cytokines and symptoms [34, 81]. Secondly, we did not control for different antipsychotic medication, as subgrouping would have created insufficient statistical power for network estimation [34, 82]. Nonetheless, all participants were prescribed antipsychotic medication. Lastly, the sample size was relatively small for network analyses and levels of inflammatory cytokines and symptoms were also lower in the current cohort compared to previous cohorts [20, 29] (with *L-1β* and *IL-4* being close to the lower limit of detection). Given the possibility of a biological gradient regarding cytokine-symptom associations in schizophrenia, the current findings might not have captured the full extent and strength of cytokine-symptom associations [15].

### Conclusions

We report that *IL-6, TNF-*α and *IFN-γ* had the greatest influence in a network of cytokines in individuals with schizophrenia. Such pro-inflammatory cytokines were connected to depressive symptoms and suspiciousness, rather than positive or negative symptoms. Networks at 6 months contained fewer cytokine and symptoms connections in those exposed to minocycline compared to the placebo group. Thus, *IL-6, TNF-*α and *IFN-γ* might represent important biomarkers for targeted treatments in schizophrenia and depression might represent an inflammatory endophenotype of schizophrenia with potential responsiveness to immune-based therapies. Targeted experimental approaches and precision medicine studies focused on *IL-6* and related cytokines within subjects with a depressive phenotypic profile are now required to test whether these are clinically meaningful effects. However, given the paucity of novel treatments for schizophrenia and enduring poor outcomes, this approach offers considerable potential for advancement in the field.

### Supplementary information

Supplementary Materials

## References

[1] Rossler W, Salize HJ, Van Os J, Riecher-Rössler A (2005). Size of burden of schizophrenia and psychotic disorders. Eur Neuropsychopharmacol.

[2] Jääskeläinen E, Juola P, Hirvonen N, McGrath JJ, Saha S, Isohanni M (2013). A systematic review and meta-analysis of recovery in schizophrenia. Schizophr Bull.

[3] Upthegrove R, Manzanares-Teson N, Barnes NM (2014). Cytokine function in medication-naive first episode psychosis: a systematic review and meta-analysis. Schizophr Res.

[4] Miller BJ, Buckley P, Seabolt W, Mellor A, Kirkpatrick B (2011). Meta-analysis of cytokine alterations in schizophrenia: clinical status and antipsychotic effects. Biol Psychiatry.

[5] Momtazmanesh S, Zare-Shahabadi A, Rezaei N (2019). Cytokine alterations in schizophrenia: an updated review. Front Psychiatry.

[6] Potvin S, Stip E, Sepehry AA, Gendron A, Bah R, Kouassi E (2008). Inflammatory cytokine alterations in schizophrenia: a systematic quantitative review. Biol Psychiatry.

[7] Capuzzi E, Bartoli F, Crocamo C, Clerici M, Carrà G (2017). Acute variations of cytokine levels after antipsychotic treatment in drug-naïve subjects with a first-episode psychosis: a meta-analysis. Neurosci Biobehav Rev.

[8] Goldsmith DR, Rapaport MH, Miller BJ (2016). A meta-analysis of blood cytokine network alterations in psychiatric patients: comparisons between schizophrenia, bipolar disorder and depression. Mol Psychiatry.

[9] Goldsmith DR, Haroon E, Miller AH, Strauss GP, Buckley PF, Miller BJ (2018). TNF-α and IL-6 are associated with the deficit syndrome and negative symptoms in patients with chronic schizophrenia. Schizophr Res.

[10] Fillman SG, Sinclair D, Fung SJ, Webster MJ, Shannon Weickert C (2014). Markers of inflammation and stress distinguish subsets of individuals with schizophrenia and bipolar disorder. Transl Psychiatry.

[11] Bergink V, Gibney SM, Drexhage HA (2014). Autoimmunity, inflammation, and psychosis: a search for peripheral markers. Biol psychiatry.

[12] Noto C, Ota VK, Santoro ML, Ortiz BB, Rizzo LB, Higuchi CH (2015). Effects of depression on the cytokine profile in drug naive first-episode psychosis. Schizophr Res.

[13] Müller N (2011). Inflammation and schizophrenia: pathophysiological and therapeutic aspects. Minerva Psichiatr.

[14] Kim YK, Myint AM, Lee BH, Han CS, Lee HJ, Kim DJ (2004). Th1, Th2 and Th3 cytokine alteration in schizophrenia. Prog Neuropsychopharmacol Biol Psychiatry.

[15] Miller BJ, Goldsmith DR (2019). Inflammatory biomarkers in schizophrenia: Implications for heterogeneity and neurobiology. Biomark Neuropsychiatry.

[16] Khandaker GM, Pearson RM, Zammit S, Lewis G, Jones PB (2014). Association of serum interleukin 6 and C-reactive protein in childhood with depression and psychosis in young adult life: a population-based longitudinal study. JAMA Psychiatry.

[17] Dean B (2011). Understanding the role of inflammatory-related pathways in the pathophysiology and treatment of psychiatric disorders: evidence from human peripheral studies and CNS studies. Int J Neuropsychopharmacol.

[18] Kowalski J, Blada P, Kucia K, Madej A, Herman ZS (2001). Neuroleptics normalize increased release of interleukin-1β and tumor necrosis factor-α from monocytes in schizophrenia. Schizophr Res.

[19] Pillinger T, Osimo EF, Brugger S, Mondelli V, McCutcheon RA, Howes OD (2019). A meta-analysis of immune parameters, variability, and assessment of modal distribution in psychosis and test of the immune subgroup hypothesis. Schizophr Bull.

[20] Krynicki CR, Dazzan P, Pariante CM, Barnes NM, Vincent RC, Roberts A (2021). Deconstructing depression and negative symptoms of schizophrenia; differential and longitudinal immune correlates, and response to minocycline treatment. Brain Behav Immun.

[21] Goldsmith DR, Haroon E, Miller AH, Addington J, Bearden C, Cadenhead K (2019). Association of baseline inflammatory markers and the development of negative symptoms in individuals at clinical high risk for psychosis. Brain Behav Immun.

[22] Asevedo E, Rizzo LB, Gadelha A, Mansur RB, Ota VK, Berberian AA (2014). Peripheral interleukin-2 level is associated with negative symptoms and cognitive performance in schizophrenia. Physiol Behav.

[23] El Kissi Y, Samoud S, Mtiraoui A, Letaief L, Hannachi N, Ayachi M (2015). Increased Interleukin-17 and decreased BAFF serum levels in drug-free acute schizophrenia. Psychiatry Res.

[24] Garcia-Rizo C, Fernandez-Egea E, Oliveira C, Justicia A, Bernardo M, Kirkpatrick B (2012). Inflammatory markers in antipsychotic-naïve patients with nonaffective psychosis and deficit vs. nondeficit features. Psychiatry Res.

[25] Liu H, Kang Y, Liang J, Li C, Xiu M, Chen D (2012). Lower serum interleukin-2 levels in schizophrenic patients with tardive dyskinesia. Psychiatry Res.

[26] Noto C, Maes M, Ota VK, Teixeira AL, Bressan RA, Gadelha A (2015). High predictive value of immune-inflammatory biomarkers for schizophrenia diagnosis and association with treatment resistance. World J Biol Psychiatry.

[27] Noto CS, Gadelha A, Belangero SI, Smith MAC, de Aguiar BW, Panizzuti B (2011). Association of biomarkers and depressive symptoms in schizophrenia. Neurosci Lett.

[28] Jeppesen R, Christensen RH, Pedersen EM, Nordentoft M, Hjorthøj C, Köhler-Forsberg O (2020). Efficacy and safety of anti-inflammatory agents in treatment of psychotic disorders—a comprehensive systematic review and meta-analysis. Brain Behav Immun.

[29] Çakici N, Van Beveren NJM, Judge-Hundal G, Koola MM, Sommer IEC (2019). An update on the efficacy of anti-inflammatory agents for patients with schizophrenia: a meta-analysis. Psychol Med.

[30] Sommer IE, van Westrhenen R, Begemann MJ, de Witte LD, Leucht S, Kahn RS (2014). Efficacy of anti-inflammatory agents to improve symptoms in patients with schizophrenia: an update. Schizophr Bull.

[31] Nettis MA, Lombardo G, Hastings C, Zajkowska Z, Mariani N, Nikkheslat N (2021). Augmentation therapy with minocycline in treatment-resistant depression patients with low-grade peripheral inflammation: results from a double-blind randomised clinical trial. Neuropsychopharmacology.

[32] Deakin B, Suckling J, Barnes TR, Byrne K, Chaudhry IB, Dazzan P (2018). The benefit of minocycline on negative symptoms of schizophrenia in patients with recent-onset psychosis (BeneMin): a randomised, double-blind, placebo-controlled trial. lancet Psychiatry.

[33] Schett G, Elewaut D, McInnes IB, Dayer JM, Neurath MF (2013). How cytokine networks fuel inflammation: toward a cytokine-based disease taxonomy. Nat Med.

[34] Fried EI, Von Stockert S, Haslbeck JMB, Lamers F, Schoevers RA, Penninx BWJH (2020). Using network analysis to examine links between individual depressive symptoms, inflammatory markers, and covariates. Psychol Med.

[35] Lamers F, Vogelzangs N, Merikangas KR, De Jonge P, Beekman ATF, Penninx BWJH (2013). Evidence for a differential role of HPA-axis function, inflammation and metabolic syndrome in melancholic versus atypical depression. Mol Psychiatry.

[36] Kim YK, Na KS, Shin KH, Jung HY, Choi SH, Kim JB (2007). Cytokine imbalance in the pathophysiology of major depressive disorder. Prog Neuropsychopharmacol Biol Psychiatry.

[37] Eller T, Vasar V, Shlik J, Maron E (2008). Pro-inflammatory cytokines and treatment response to escitalopram in major depressive disorder. Prog Neuropsychopharmacol Biol Psychiatry.

[38] Milaneschi Y, Lamers F, Berk M, Penninx BW (2020). Depression heterogeneity and its biological underpinnings: toward immunometabolic depression. Biol Psychiatry.

[39] Goldsmith DR, Haroon E, Woolwine BJ, Jung MY, Wommack EC, Harvey PD (2016). Inflammatory markers are associated with decreased psychomotor speed in patients with major depressive disorder. Brain Behav Immun.

[40] Felger JC, Haroon E, Patel TA, Goldsmith DR, Wommack EC, Woolwine BJ (2020). What does plasma CRP tell us about peripheral and central inflammation in depression?. Mol Psychiatry.

[41] Vollmer-Conna UA, Fazou C, Cameron B, Li H, Brennan C, Luck L (2004). Production of pro-inflammatory cytokines correlates with the symptoms of acute sickness behaviour in humans. Psychol Med.

[42] Dantzer R, O'connor JC, Freund GG, Johnson RW, Kelley KW (2008). From inflammation to sickness and depression: when the immune system subjugates the brain. Nat Rev Neurosci.

[43] Herniman SE, Allott K, Phillips LJ, Wood SJ, Uren J, Mallawaarachchi SR (2019). Depressive psychopathology in first-episode schizophrenia spectrum disorders: a systematic review, meta-analysis and meta-regression. Psychol Med.

[44] Lisiecka DM, Suckling J, Barnes TR, Chaudhry IB, Dazzan P, Husain N (2015). The benefit of minocycline on negative symptoms in early-phase psychosis in addition to standard care-extent and mechanism (BeneMin): study protocol for a randomised controlled trial. Trials.

[45] American Psychiatric Association. Diagnostic and statistical manual of mental disorders (4th edn). Washington, DC: American Psychiatric Association; 1994.

[46] Sheehan DV, Lecrubier Y, Sheehan KH, Amorim P, Janavs J, Weiller E (1998). The Mini-International Neuropsychiatric Interview (MINI): The development and validation of a structured diagnostic psychiatric interview for DSM-IV and ICD-10. J Clin Psychiatry.

[47] Nettis MA, Veronese M, Nikkheslat N, Mariani N, Lombardo G, Sforzini L (2020). PET imaging shows no changes in TSPO brain density after IFN-α immune challenge in healthy human volunteers. Transl Psychiatry.

[48] Russell A, Hepgul N, Nikkheslat N, Borsini A, Zajkowska Z, Moll N (2019). Persistent fatigue induced by interferon-alpha: a novel, inflammation-based, proxy model of chronic fatigue syndrome. Psychoneuroendocrinology.

[49] Kay SR, Fiszbein A, Opler LA (1987). The positive and negative syndrome scale (PANSS) for schizophrenia. Schizophr Bull.

[50] Addington D, Addington J, Schissel B (1990). A depression rating scale for schizophrenics. Schizophr Res.

[51] Lako IM, Bruggeman R, Knegtering H, Wiersma D, Schoevers RA, Slooff CJ (2012). A systematic review of instruments to measure depressive symptoms in patients with schizophrenia. J Affect Disord.

[52] Troyanskaya O, Cantor M, Sherlock G, Brown P, Hastie T, Tibshirani R (2001). Missing value estimation methods for DNA microarrays. Bioinformatics.

[53] Tabachnick, B.G., & Fidell, L.S. Using multivariate statistics (6th edn). Boston, MA: Pearson; 2013.

[54] Epskamp S, Borsboom D, Fried EI (2018). Estimating psychological networks and their accuracy: a tutorial paper. Behav Res Methods.

[55] Epskamp S, Fried EI (2018). A tutorial on regularized partial correlation networks. Psychol Methods.

[56] Tibshirani R (1996). Regression shrinkage and selection via the lasso. J R Stat Soc Ser B (Methodol).

[57] Chen J, Chen Z (2008). Extended Bayesian information criteria for model selection with large model spaces. Biometrika.

[58] Herniman SE, Phillips LJ, Wood SJ, Cotton SM, Liemburg EJ, Allott KA (2021). Interrelationships between depressive symptoms and positive and negative symptoms of recent onset schizophrenia spectrum disorders: a network analytical approach. J Psychiatr Res.

[59] Jones PJ, Ma R, McNally RJ (2019). Bridge centrality: a network approach to understanding comorbidity. Multivar Behav Res.

[60] Fried, E.I. R Tutorial: how to identify communities of items in networks. 2016. https://psych-networks.com/r-tutorial-identify-communities-items-networks/

[61] van Borkulo C, Boschloo L, Borsboom D, Penninx BWJH, Waldorp LJ, Schoevers RA (2015). Association of symptom network structure with the course of depression. JAMA Psychiatry.

[62] Terluin B, De Boer MR, De Vet HC (2016). Differences in connection strength between mental symptoms might be explained by differences in variance: Reanalysis of network data did not confirm staging. PLoS ONE.

[63] Boschloo L, Bekhuis E, Weitz ES, Reijnders M, DeRubeis RJ, Dimidjian S (2019). The symptom‐specific efficacy of antidepressant medication vs. cognitive behavioral therapy in the treatment of depression: Results from an individual patient data meta‐analysis. World Psychiatry.

[64] Ligthart S, Vaez A, Võsa U, Stathopoulou MG, de Vries PS, Prins BP (2018). Genome-wide association analyses of> 200,000 individuals identify 58 genetic loci for chronic inflammation and highlights pathways that link inflammation and complex disorders. Am J Hum Genet.

[65] Hartwig FP, Borges MC, Horta BL, Bowden J, Smith GD (2017). Inflammatory biomarkers and risk of schizophrenia: a 2-sample mendelian randomization study. JAMA Psychiatry.

[66] Khandaker GM, Zammit S, Burgess S, Lewis G, Jones PB (2018). Association between a functional interleukin 6 receptor genetic variant and risk of depression and psychosis in a population-based birth cohort. Brain Behav Immun.

[67] Borsboom D (2017). A network theory of mental disorders. World Psychiatry.

[68] Myin-Germeys I, van Os J (2007). Stress-reactivity in psychosis: evidence for an affective pathway to psychosis. Clin Psychol Rev.

[69] Berk M, Williams LJ, Jacka FN, O’Neil A, Pasco JA, Moylan S (2013). So depression is an inflammatory disease, but where does the inflammation come from?. BMC Med.

[70] Pelizza L, Azzali S, Garlassi S, Paterlini F, Scazza I, Chiri LR (2018). Adolescents at ultra-high risk of psychosis in Italian neuropsychiatry services: prevalence, psychopathology and transition rate. Eur Child Adolesc Psychiatry.

[71] Spada G, Molteni S, Pistone C, Chiappedi M, McGuire P, Fusar-Poli P (2016). Identifying children and adolescents at ultra high risk of psychosis in Italian neuropsychiatry services: a feasibility study. Eur Child Adolesc Psychiatry.

[72] Gong K, Zou X, Fuchs PN, Lin Q (2015). Minocycline inhibits neurogenic inflammation by blocking the effects of tumor necrosis factor‐α. Clin Exp Pharmacol Physiol.

[73] Husain MI, Chaudhry IB, Husain N, Khoso AB, Rahman RR, Hamirani MM (2017). Minocycline as an adjunct for treatment-resistant depressive symptoms: a pilot randomised placebo-controlled trial. J Psychopharmacol.

[74] Oya K, Kishi T, Iwata N (2014). Efficacy and tolerability of minocycline augmentation therapy in schizophrenia: a systematic review and meta‐analysis of randomized controlled trials. Hum Psychopharmacol: Clin Exp.

[75] Solmi M, Veronese N, Thapa N, Facchini S, Stubbs B, Fornaro M (2017). Systematic review and meta-analysis of the efficacy and safety of minocycline in schizophrenia. CNS Spectr.

[76] Birchwood M (2003). Pathways to emotional dysfunction in first-episode psychosis. Br J Psychiatry.

[77] Watson PWB, Garety PA, Weinman J, Dunn G, Bebbington PE, Fowler D (2006). Emotional dysfunction in schizophrenia spectrum psychosis: the role of illness perceptions. Psychol Med.

[78] Marwaha S, Broome MR, Bebbington PE, Kuipers E, Freeman D (2014). Mood instability and psychosis: analyses of British national survey data. Schizophr Bull.

[79] Upthegrove R, Broome MR, Caldwell K, Ives J, Oyebode F, Wood SJ (2016). Understanding auditory verbal hallucinations: a systematic review of current evidence. Acta Psychiatr Scand.

[80] Betz LT, Penzel N, Kambeitz-Ilankovic L, Rosen M, Chisholm K, Stainton A (2020). General psychopathology links burden of recent life events and psychotic symptoms in a network approach. NPJ Schizophr.

[81] Mac Giollabhui N, Ellman LM, Coe CL, Byrne ML, Abramson LY, Alloy LB (2020). To exclude or not to exclude: considerations and recommendations for C-reactive protein values higher than 10 mg/L. Brain Behav Immun.

[82] O’Connor MF, Bower JE, Cho HJ, Creswell JD, Dimitrov S, Hamby ME (2009). To assess, to control, to exclude: effects of biobehavioral factors on circulating inflammatory markers. Brain Behav Immun.

