# Supplementary material for: Network analysis of inflammation and symptoms in recent onset schizophrenia and the influence of minocycline during a clinical trial
Source: Transl Psychiatry. 2023 Sep 18;13:297. doi: 10.1038/s41398-023-02570-8 (PMC10507090; doi:10.1038/s41398-023-02570-8)
Supplement: Supplementary file 1 — Supplementary Materials [file 41398_2023_2570_MOESM1_ESM.pdf]

Supplementary materials for baseline network of inflammatory cytokines

Nonparametric bootstrapped confidence intervals. The Figure below presents the nonparametric bootstrapped confidence intervals of estimated edge-weights for the network comprising all 12 inflammatory cytokines. As can be seen, there were wide bootstrapped CIs around the estimated edge-weights, meaning that the relative strength of edge-weights should be interpreted with care.

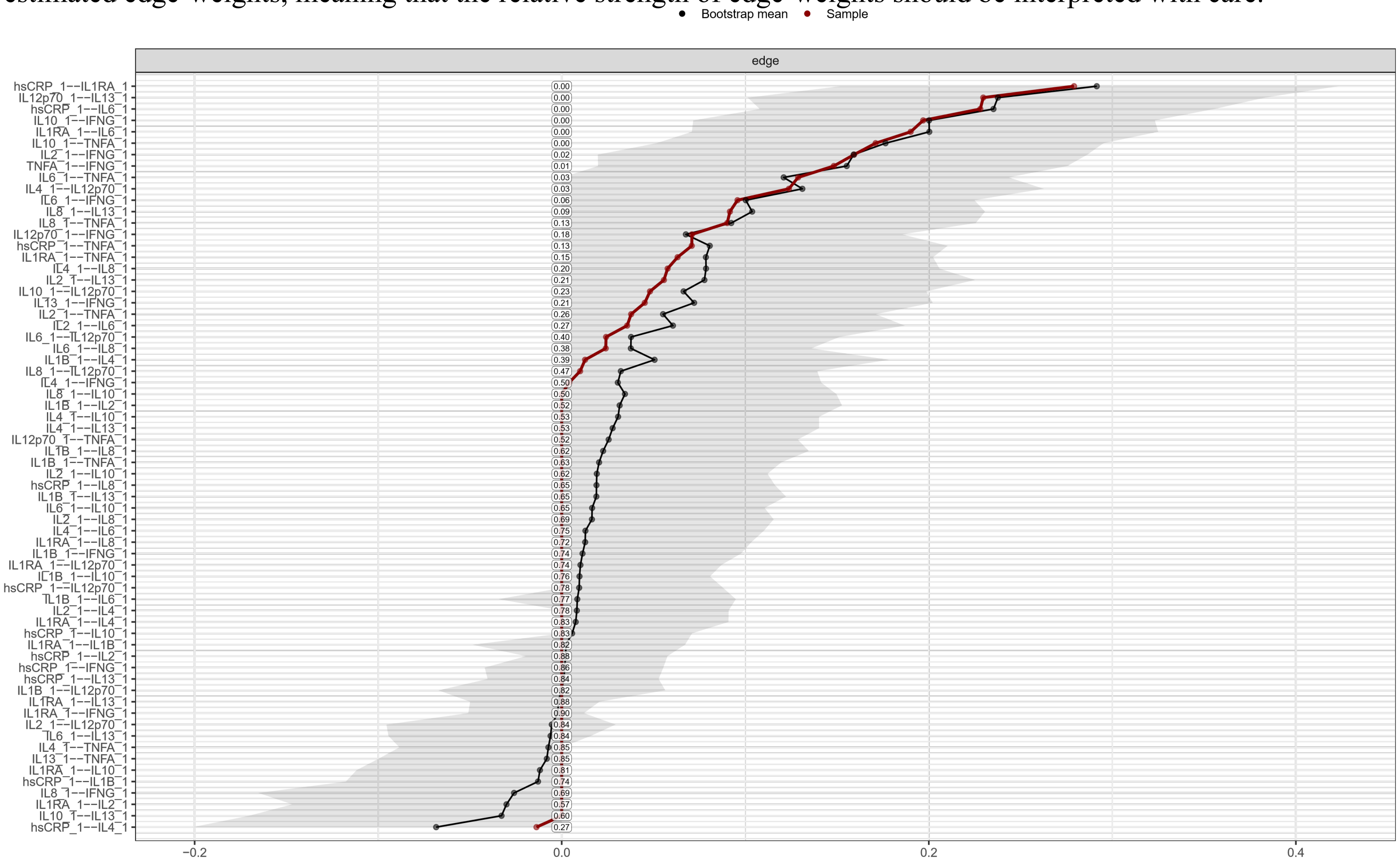

Notes. The red line indicates the sample values and they grey area indicates the bootstrapped CIs. Each horizontal line represents one edge of the network, ordered from the edge with the highest edge-weight to the edge weight with the lowest edge-weight. In the case of equivalent sampled edge weights, the mean of the bootstrap samples was used in ordering the edges.

Case-dropping bootstrapping Procedures. The Figures below show the CS-coefficients for edge weights and strength centrality for the network comprising all 12 inflammatory cytokines. CS-co-efficient were 0.52 and 0.44, respectively.

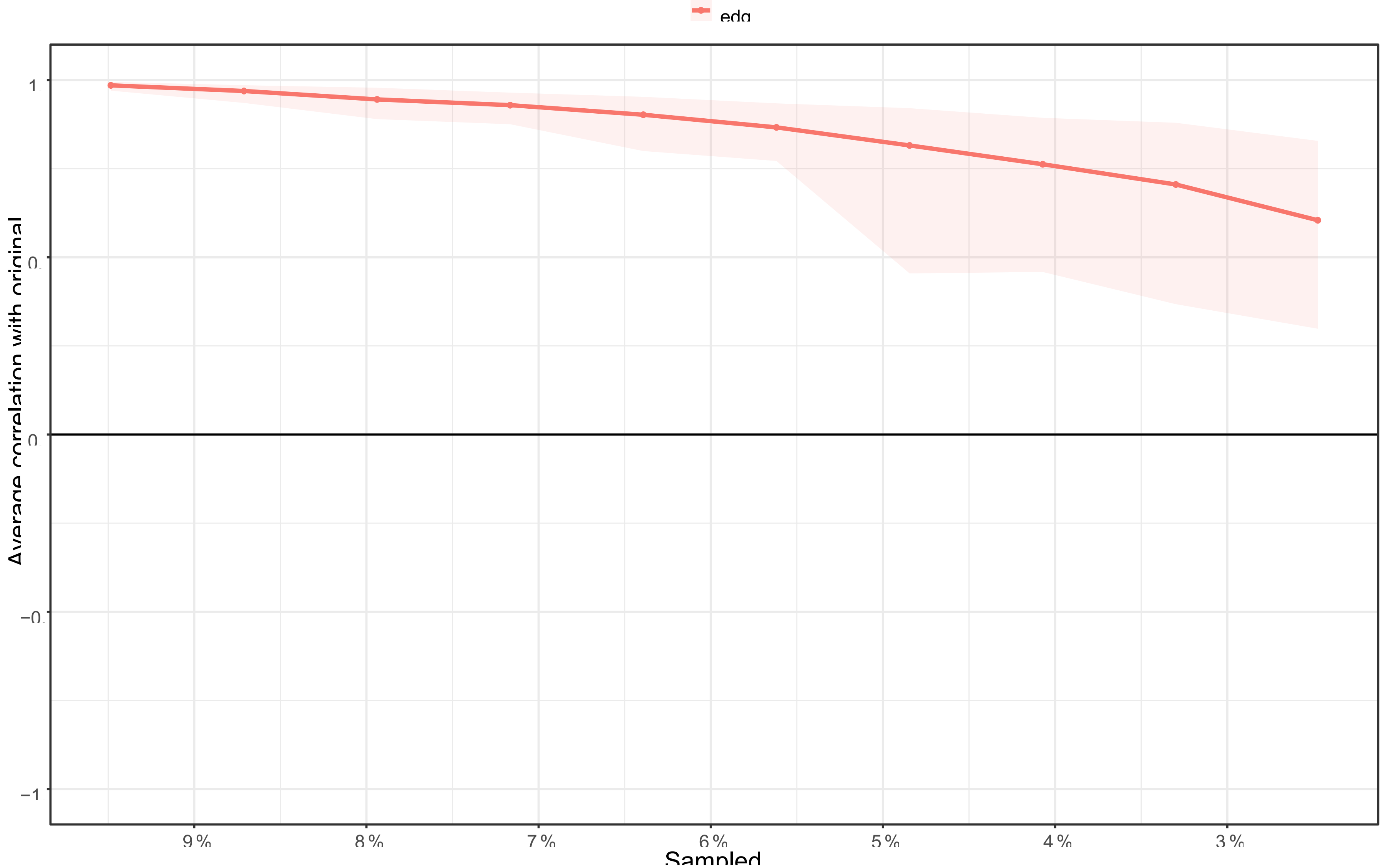

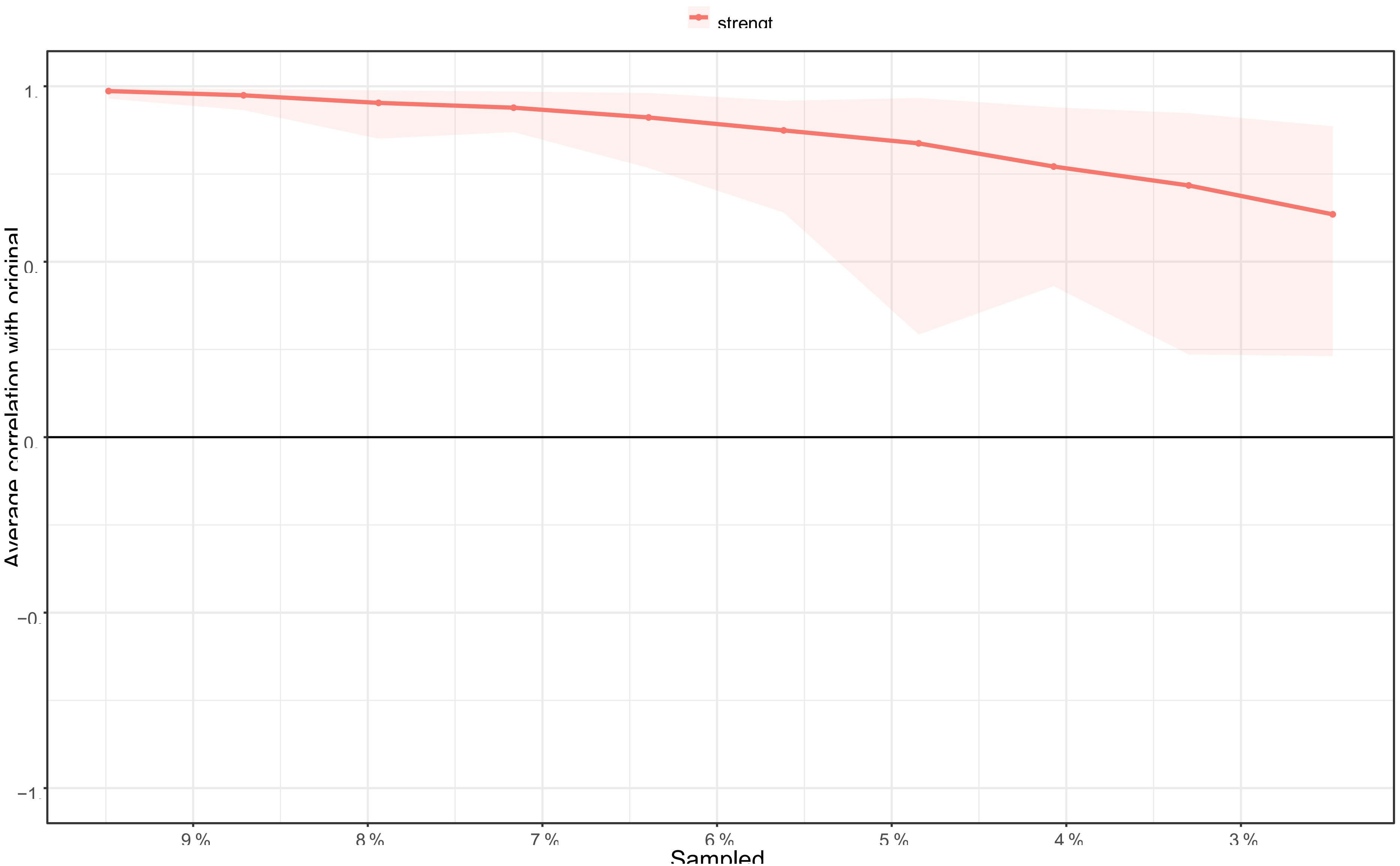
strengt
1.
0.
0.
−0.
−1.
Average correlation with original
9 %
8 %
7 %
6 %
5 %
4 %
3 %
Sampled

Supplementary materials for network of inflammatory cytokines and symptoms at baseline

Nonparametric bootstrapped confidence intervals. The Figure below presents the nonparametric bootstrapped confidence intervals of estimated edge-weights for the network comprising influential pro-inflammatory cytokines and symptoms whilst controlling for covariates. As can be seen, There were sufficiently narrow bootstrapped CIs around the estimated edge-weights. The *IL-6—self-depreciation* edge-weight was estimated to be above zero in 81% of the 1000 nonparametric bootstrapped procedures, *IFN-γ—hopelessness* in 64%, and *TNF*-α—*suspiciousness* in 63%.

Figure Notes. The red line indicates the sample values and they grey area indicates the bootstrapped CIs. Each horizontal line represents one edge of the network, ordered from the edge with the highest edge-weight to the edge weight with the lowest edge-weight. In the case of equivalent sampled edge weights, the mean of the bootstrap samples was used in ordering the edges.

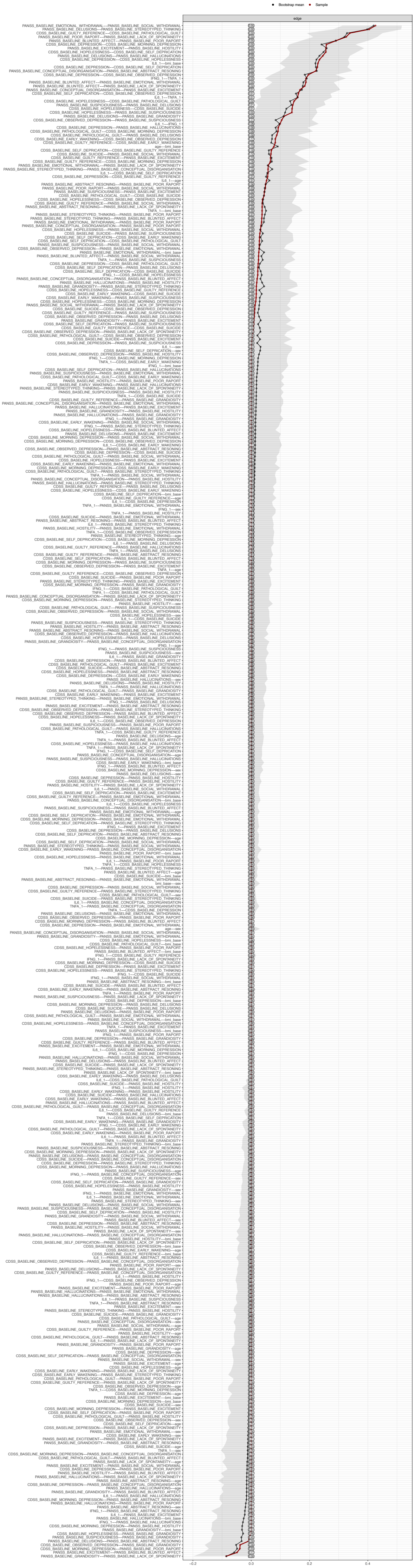
Bootstrap mean
Sample
edge
-0.2
0.0
0.2
0.4

Case-dropping bootstrapped procedures. The Figures below show the CS-coefficients for edge-weights for the network comprising influential pro-inflammatory cytokines and symptoms of the depression and suspiciousness community. The CS-co-efficient was 0.67.

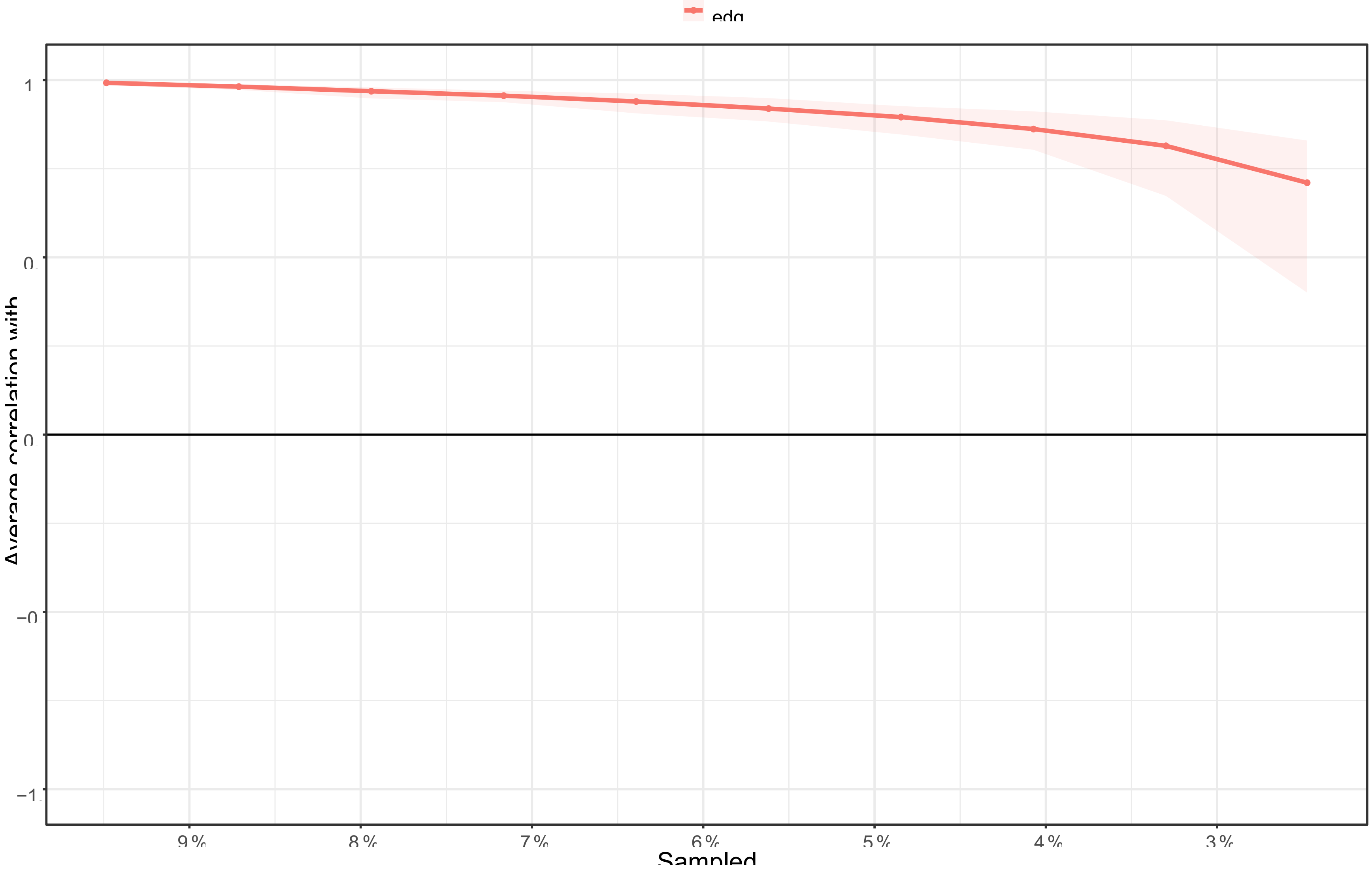

Supplementary materials for network of treatment allocation, influential pro-inflammatory cytokines and symptoms whilst controlling for covariates at 6-month follow-up

Nonparametric bootstrapped confidence intervals. The Figure below presents the nonparametric bootstrapped confidence intervals of estimated edge-weights for the network comprising treatment allocation, influential pro-inflammatory cytokines, and symptoms whilst controlling for covariates at six-month follow-up. As can be seen, there were sufficiently narrow bootstrapped CIs around the estimated edge-weights. The *treatment— TNF-α* association was estimated to be above zero in 96% of the 1,000 nonparametric bootstrapped procedures, and *treatment—early wakening* in 60%.

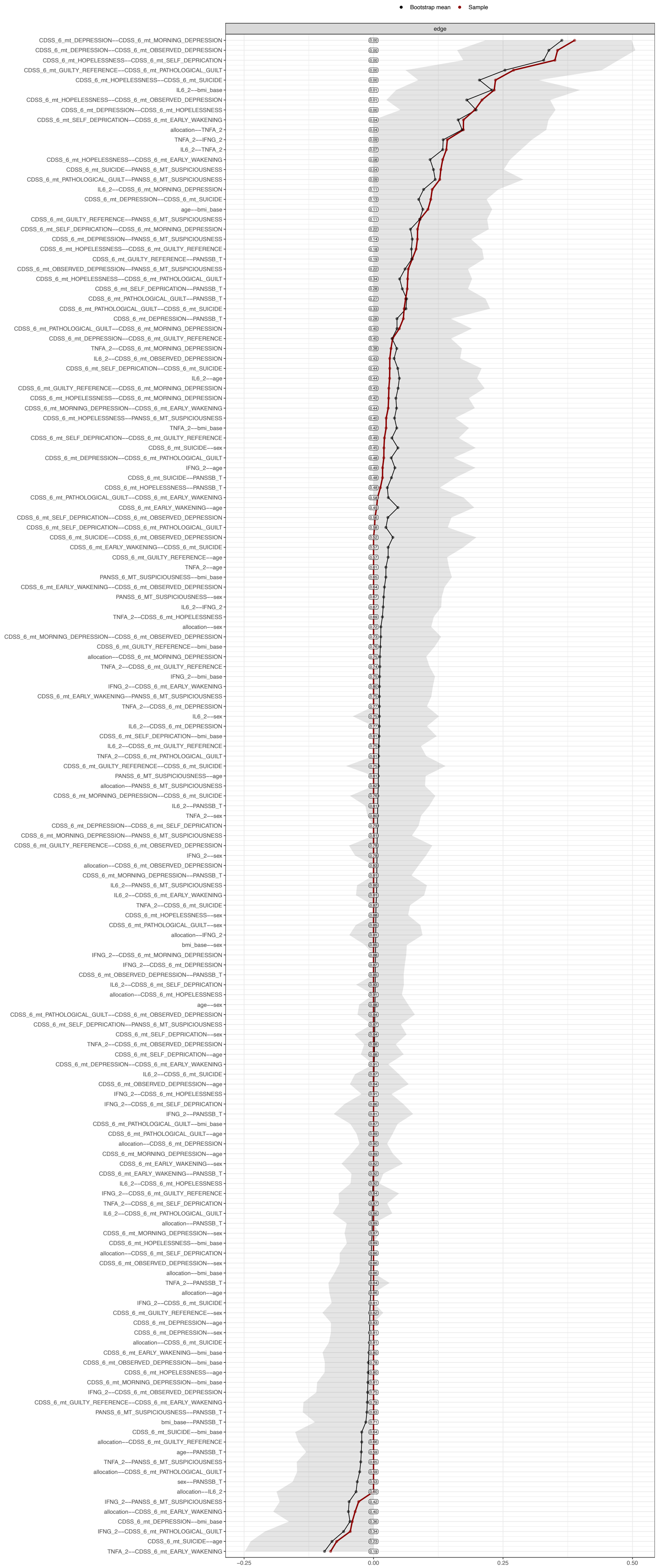

Bootstrap mean
Sample
edge
CDSS_6_mt_DEPRESSION--CDSS_6_mt_MORNING_DEPRESSION
CDSS_6_mt_DEPRESSION--CDSS_6_mt_OBSERVED_DEPRESSION
CDSS_6_mt_HOPELESSNESS--CDSS_6_mt_SELF_DEPRICATION
CDSS_6_mt_GUILTY_REFERENCE--CDSS_6_mt_PATHOLOGICAL_GUILT
CDSS_6_mt_HOPELESSNESS--CDSS_6_mt_SUICIDE
IL6_2--bmi_base
CDSS_6_mt_HOPELESSNESS--CDSS_6_mt_OBSERVED_DEPRESSION
CDSS_6_mt_DEPRESSION--CDSS_6_mt_HOPELESSNESS
CDSS_6_mt_SELF_DEPRICATION--CDSS_6_mt_EARLY_WAKENING
allocation--TNFA_2
TNFA_2--IFNG_2
IL6_2--TNFA_2
CDSS_6_mt_HOPELESSNESS--CDSS_6_mt_EARLY_WAKENING
CDSS_6_mt_SUICIDE--PANSS_6_MT_SUSPICIOUSNESS
CDSS_6_mt_PATHOLOGICAL_GUILT--PANSS_6_MT_SUSPICIOUSNESS
IL6_2--CDSS_6_mt_MORNING_DEPRESSION
CDSS_6_mt_DEPRESSION--CDSS_6_mt_SUICIDE
age--bmi_base
CDSS_6_mt_GUILTY_REFERENCE--PANSS_6_MT_SUSPICIOUSNESS
CDSS_6_mt_SELF_DEPRICATION--CDSS_6_mt_MORNING_DEPRESSION
CDSS_6_mt_DEPRESSION--PANSS_6_MT_SUSPICIOUSNESS
CDSS_6_mt_HOPELESSNESS--CDSS_6_mt_GUILTY_REFERENCE
CDSS_6_mt_GUILTY_REFERENCE--PANSSB_T
CDSS_6_mt_OBSERVED_DEPRESSION--PANSS_6_MT_SUSPICIOUSNESS
CDSS_6_mt_HOPELESSNESS--CDSS_6_mt_PATHOLOGICAL_GUILT
CDSS_6_mt_SELF_DEPRICATION--PANSSB_T
CDSS_6_mt_PATHOLOGICAL_GUILT--PANSSB_T
CDSS_6_mt_PATHOLOGICAL_GUILT--CDSS_6_mt_SUICIDE
CDSS_6_mt_DEPRESSION--PANSSB_T
CDSS_6_mt_PATHOLOGICAL_GUILT--CDSS_6_mt_MORNING_DEPRESSION
CDSS_6_mt_DEPRESSION--CDSS_6_mt_GUILTY_REFERENCE
TNFA_2--CDSS_6_mt_MORNING_DEPRESSION
IL6_2--CDSS_6_mt_OBSERVED_DEPRESSION
CDSS_6_mt_SELF_DEPRICATION--CDSS_6_mt_SUICIDE
IL6_2--age
CDSS_6_mt_GUILTY_REFERENCE--CDSS_6_mt_MORNING_DEPRESSION
CDSS_6_mt_HOPELESSNESS--CDSS_6_mt_MORNING_DEPRESSION
CDSS_6_mt_MORNING_DEPRESSION--CDSS_6_mt_EARLY_WAKENING
CDSS_6_mt_HOPELESSNESS--PANSS_6_MT_SUSPICIOUSNESS
TNFA_2--bmi_base
CDSS_6_mt_SELF_DEPRICATION--CDSS_6_mt_GUILTY_REFERENCE
CDSS_6_mt_SUICIDE--sex
CDSS_6_mt_DEPRESSION--CDSS_6_mt_PATHOLOGICAL_GUILT
IFNG_2--age
CDSS_6_mt_SUICIDE--PANSSB_T
CDSS_6_mt_HOPELESSNESS--PANSSB_T
CDSS_6_mt_PATHOLOGICAL_GUILT--CDSS_6_mt_EARLY_WAKENING
CDSS_6_mt_EARLY_WAKENING--age
CDSS_6_mt_SELF_DEPRICATION--CDSS_6_mt_OBSERVED_DEPRESSION
CDSS_6_mt_SELF_DEPRICATION--CDSS_6_mt_PATHOLOGICAL_GUILT
CDSS_6_mt_SUICIDE--CDSS_6_mt_OBSERVED_DEPRESSION
CDSS_6_mt_EARLY_WAKENING--CDSS_6_mt_SUICIDE
CDSS_6_mt_GUILTY_REFERENCE--age
TNFA_2--age
PANSS_6_MT_SUSPICIOUSNESS--bmi_base
CDSS_6_mt_EARLY_WAKENING--CDSS_6_mt_OBSERVED_DEPRESSION
PANSS_6_MT_SUSPICIOUSNESS--sex
IL6_2--IFNG_2
TNFA_2--CDSS_6_mt_HOPELESSNESS
allocation--sex
CDSS_6_mt_MORNING_DEPRESSION--CDSS_6_mt_OBSERVED_DEPRESSION
CDSS_6_mt_GUILTY_REFERENCE--bmi_base
allocation--CDSS_6_mt_MORNING_DEPRESSION
TNFA_2--CDSS_6_mt_GUILTY_REFERENCE
IFNG_2--bmi_base
IFNG_2--CDSS_6_mt_EARLY_WAKENING
CDSS_6_mt_EARLY_WAKENING--PANSS_6_MT_SUSPICIOUSNESS
TNFA_2--CDSS_6_mt_DEPRESSION
IL6_2--sex
IL6_2--CDSS_6_mt_DEPRESSION
CDSS_6_mt_SELF_DEPRICATION--bmi_base
IL6_2--CDSS_6_mt_GUILTY_REFERENCE
TNFA_2--CDSS_6_mt_PATHOLOGICAL_GUILT
CDSS_6_mt_GUILTY_REFERENCE--CDSS_6_mt_SUICIDE
PANSS_6_MT_SUSPICIOUSNESS--age
allocation--PANSS_6_MT_SUSPICIOUSNESS
CDSS_6_mt_MORNING_DEPRESSION--CDSS_6_mt_SUICIDE
IL6_2--PANSSB_T
TNFA_2--sex
CDSS_6_mt_DEPRESSION--CDSS_6_mt_SELF_DEPRICATION
CDSS_6_mt_MORNING_DEPRESSION--PANSS_6_MT_SUSPICIOUSNESS
CDSS_6_mt_GUILTY_REFERENCE--CDSS_6_mt_OBSERVED_DEPRESSION
IFNG_2--sex
allocation--CDSS_6_mt_OBSERVED_DEPRESSION
CDSS_6_mt_MORNING_DEPRESSION--PANSSB_T
IL6_2--PANSS_6_MT_SUSPICIOUSNESS
IL6_2--CDSS_6_mt_EARLY_WAKENING
TNFA_2--CDSS_6_mt_SUICIDE
CDSS_6_mt_HOPELESSNESS--sex
CDSS_6_mt_PATHOLOGICAL_GUILT--sex
allocation--IFNG_2
bmi_base--sex
IFNG_2--CDSS_6_mt_MORNING_DEPRESSION
IFNG_2--CDSS_6_mt_DEPRESSION
CDSS_6_mt_OBSERVED_DEPRESSION--PANSSB_T
IL6_2--CDSS_6_mt_SELF_DEPRICATION
allocation--CDSS_6_mt_HOPELESSNESS
age--sex
CDSS_6_mt_PATHOLOGICAL_GUILT--CDSS_6_mt_OBSERVED_DEPRESSION
CDSS_6_mt_SELF_DEPRICATION--PANSS_6_MT_SUSPICIOUSNESS
CDSS_6_mt_SELF_DEPRICATION--sex
TNFA_2--CDSS_6_mt_OBSERVED_DEPRESSION
CDSS_6_mt_SELF_DEPRICATION--age
CDSS_6_mt_DEPRESSION--CDSS_6_mt_EARLY_WAKENING
IL6_2--CDSS_6_mt_SUICIDE
CDSS_6_mt_OBSERVED_DEPRESSION--age
IFNG_2--CDSS_6_mt_HOPELESSNESS
IFNG_2--CDSS_6_mt_SELF_DEPRICATION
IFNG_2--PANSSB_T
CDSS_6_mt_PATHOLOGICAL_GUILT--bmi_base
CDSS_6_mt_PATHOLOGICAL_GUILT--age
allocation--CDSS_6_mt_DEPRESSION
CDSS_6_mt_MORNING_DEPRESSION--age
CDSS_6_mt_EARLY_WAKENING--sex
CDSS_6_mt_EARLY_WAKENING--PANSSB_T
IL6_2--CDSS_6_mt_HOPELESSNESS
IFNG_2--CDSS_6_mt_GUILTY_REFERENCE
TNFA_2--CDSS_6_mt_SELF_DEPRICATION
IL6_2--CDSS_6_mt_PATHOLOGICAL_GUILT
allocation--PANSSB_T
CDSS_6_mt_MORNING_DEPRESSION--sex
CDSS_6_mt_HOPELESSNESS--bmi_base
allocation--CDSS_6_mt_SELF_DEPRICATION
CDSS_6_mt_OBSERVED_DEPRESSION--sex
allocation--bmi_base
TNFA_2--PANSSB_T
allocation--age
IFNG_2--CDSS_6_mt_SUICIDE
CDSS_6_mt_GUILTY_REFERENCE--sex
CDSS_6_mt_DEPRESSION--age
CDSS_6_mt_DEPRESSION--sex
allocation--CDSS_6_mt_SUICIDE
CDSS_6_mt_EARLY_WAKENING--bmi_base
CDSS_6_mt_OBSERVED_DEPRESSION--bmi_base
CDSS_6_mt_HOPELESSNESS--age
CDSS_6_mt_MORNING_DEPRESSION--bmi_base
IFNG_2--CDSS_6_mt_OBSERVED_DEPRESSION
CDSS_6_mt_GUILTY_REFERENCE--CDSS_6_mt_EARLY_WAKENING
PANSS_6_MT_SUSPICIOUSNESS--PANSSB_T
bmi_base--PANSSB_T
CDSS_6_mt_SUICIDE--bmi_base
allocation--CDSS_6_mt_GUILTY_REFERENCE
age--PANSSB_T
TNFA_2--PANSS_6_MT_SUSPICIOUSNESS
allocation--CDSS_6_mt_PATHOLOGICAL_GUILT
sex--PANSSB_T
allocation--IL6_2
IFNG_2--PANSS_6_MT_SUSPICIOUSNESS
allocation--CDSS_6_mt_EARLY_WAKENING
CDSS_6_mt_DEPRESSION--bmi_base
IFNG_2--CDSS_6_mt_PATHOLOGICAL_GUILT
CDSS_6_mt_SUICIDE--age
TNFA_2--CDSS_6_mt_EARLY_WAKENING
-0.25
0.00
0.25
0.50

Case-dropping bootstrapped procedures. The Figures below show the CS-coefficients for edge-weights for the network comprising treatment allocation, influential pro-inflammatory cytokines, and symptoms of the depression and suspiciousness community. The CS-co-efficient was 0.52.

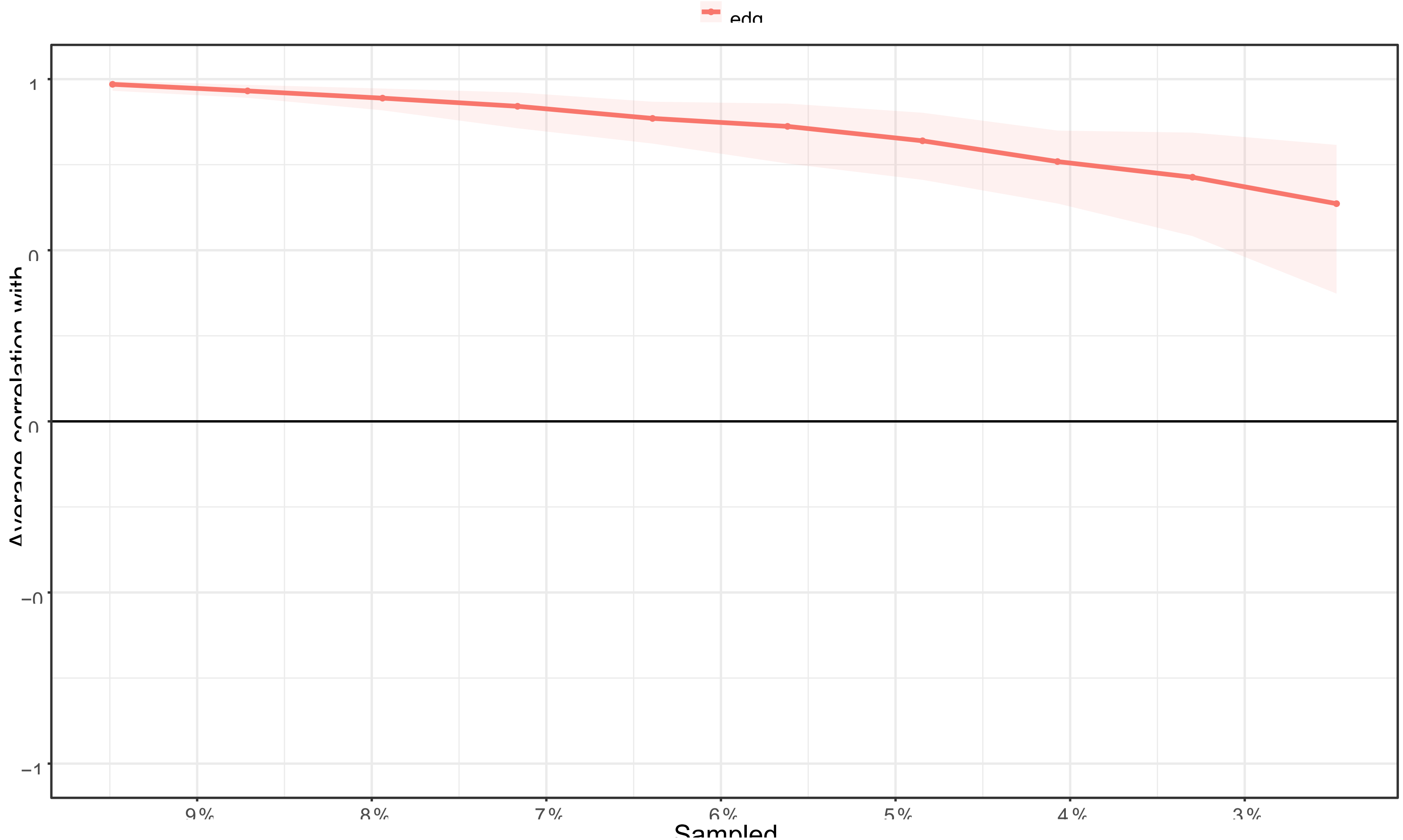

Supplementary materials for network of influential pro-inflammatory cytokines and symptoms whilst controlling for covariates at 6-month follow-up in PLACEBO group

Nonparametric bootstrapped confidence intervals. The Figure below presents the nonparametric bootstrapped confidence intervals of estimated edge-weights for the network comprising influential pro-inflammatory cytokines and symptoms of the depression and suspiciousness community whilst controlling for covariates in the placebo group. As can be seen, there were wide bootstrapped CIs around the estimated edge-weights, suggesting that relative edge strength should be interpreted with caution. Nonetheless, the *TNF-α—IL6* association was estimated to be above zero in 98% of the 1000 nonparametric bootstrapped procedures, *TNF-α— IFN-γ* in 97%, and *IL6—IFN-γ* in 35%.

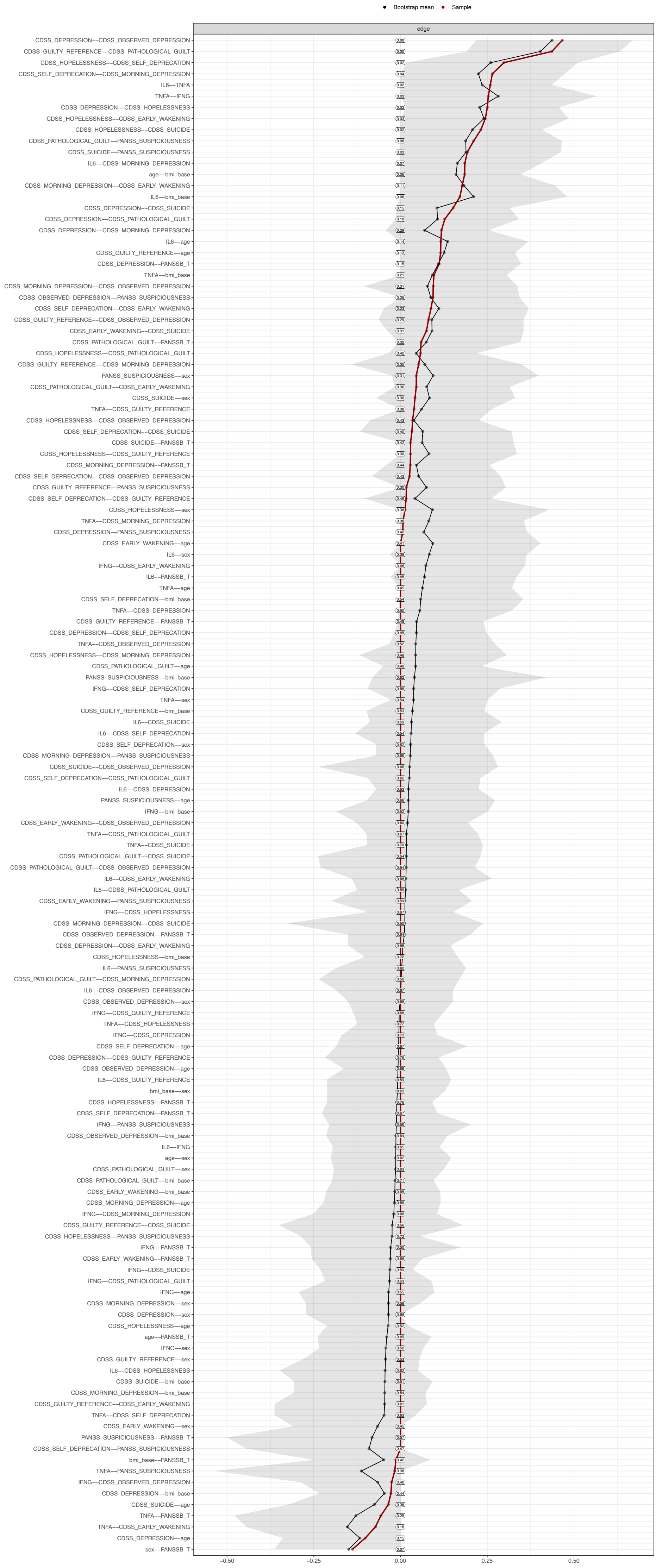

Bootstrap mean
Sample
edge
CDSS_DEPRESSION--CDSS_OBSERVED_DEPRESSION
CDSS_GUILTY_REFERENCE--CDSS_PATHOLOGICAL_GUILT
CDSS_HOPELESSNESS--CDSS_SELF_DEPRECATION
CDSS_SELF_DEPRECATION--CDSS_MORNING_DEPRESSION
IL6--TNFA
TNFA--IFNG
CDSS_DEPRESSION--CDSS_HOPELESSNESS
CDSS_HOPELESSNESS--CDSS_EARLY_WAKENING
CDSS_HOPELESSNESS--CDSS_SUICIDE
CDSS_PATHOLOGICAL_GUILT--PANSS_SUSPICIOUSNESS
CDSS_SUICIDE--PANSS_SUSPICIOUSNESS
IL6--CDSS_MORNING_DEPRESSION
age--bmi_base
CDSS_MORNING_DEPRESSION--CDSS_EARLY_WAKENING
IL6--bmi_base
CDSS_DEPRESSION--CDSS_SUICIDE
CDSS_DEPRESSION--CDSS_PATHOLOGICAL_GUILT
CDSS_DEPRESSION--CDSS_MORNING_DEPRESSION
IL6--age
CDSS_GUILTY_REFERENCE--age
CDSS_DEPRESSION--PANSSB_T
TNFA--bmi_base
CDSS_MORNING_DEPRESSION--CDSS_OBSERVED_DEPRESSION
CDSS_OBSERVED_DEPRESSION--PANSS_SUSPICIOUSNESS
CDSS_SELF_DEPRECATION--CDSS_EARLY_WAKENING
CDSS_GUILTY_REFERENCE--CDSS_OBSERVED_DEPRESSION
CDSS_EARLY_WAKENING--CDSS_SUICIDE
CDSS_PATHOLOGICAL_GUILT--PANSSB_T
CDSS_HOPELESSNESS--CDSS_PATHOLOGICAL_GUILT
CDSS_GUILTY_REFERENCE--CDSS_MORNING_DEPRESSION
PANSS_SUSPICIOUSNESS--sex
CDSS_PATHOLOGICAL_GUILT--CDSS_EARLY_WAKENING
CDSS_SUICIDE--sex
TNFA--CDSS_GUILTY_REFERENCE
CDSS_HOPELESSNESS--CDSS_OBSERVED_DEPRESSION
CDSS_SELF_DEPRECATION--CDSS_SUICIDE
CDSS_SUICIDE--PANSSB_T
CDSS_HOPELESSNESS--CDSS_GUILTY_REFERENCE
CDSS_MORNING_DEPRESSION--PANSSB_T
CDSS_SELF_DEPRECATION--CDSS_OBSERVED_DEPRESSION
CDSS_GUILTY_REFERENCE--PANSS_SUSPICIOUSNESS
CDSS_SELF_DEPRECATION--CDSS_GUILTY_REFERENCE
CDSS_HOPELESSNESS--sex
TNFA--CDSS_MORNING_DEPRESSION
CDSS_DEPRESSION--PANSS_SUSPICIOUSNESS
CDSS_EARLY_WAKENING--age
IL6--sex
IFNG--CDSS_EARLY_WAKENING
IL6--PANSSB_T
TNFA--age
CDSS_SELF_DEPRECATION--bmi_base
TNFA--CDSS_DEPRESSION
CDSS_GUILTY_REFERENCE--PANSSB_T
CDSS_DEPRESSION--CDSS_SELF_DEPRECATION
TNFA--CDSS_OBSERVED_DEPRESSION
CDSS_HOPELESSNESS--CDSS_MORNING_DEPRESSION
CDSS_PATHOLOGICAL_GUILT--age
PANSS_SUSPICIOUSNESS--bmi_base
IFNG--CDSS_SELF_DEPRECATION
TNFA--sex
CDSS_GUILTY_REFERENCE--bmi_base
IL6--CDSS_SUICIDE
IL6--CDSS_SELF_DEPRECATION
CDSS_SELF_DEPRECATION--sex
CDSS_MORNING_DEPRESSION--PANSS_SUSPICIOUSNESS
CDSS_SUICIDE--CDSS_OBSERVED_DEPRESSION
CDSS_SELF_DEPRECATION--CDSS_PATHOLOGICAL_GUILT
IL6--CDSS_DEPRESSION
PANSS_SUSPICIOUSNESS--age
IFNG--bmi_base
CDSS_EARLY_WAKENING--CDSS_OBSERVED_DEPRESSION
TNFA--CDSS_PATHOLOGICAL_GUILT
TNFA--CDSS_SUICIDE
CDSS_PATHOLOGICAL_GUILT--CDSS_SUICIDE
CDSS_PATHOLOGICAL_GUILT--CDSS_OBSERVED_DEPRESSION
IL6--CDSS_EARLY_WAKENING
IL6--CDSS_PATHOLOGICAL_GUILT
CDSS_EARLY_WAKENING--PANSS_SUSPICIOUSNESS
IFNG--CDSS_HOPELESSNESS
CDSS_MORNING_DEPRESSION--CDSS_SUICIDE
CDSS_OBSERVED_DEPRESSION--PANSSB_T
CDSS_DEPRESSION--CDSS_EARLY_WAKENING
CDSS_HOPELESSNESS--bmi_base
IL6--PANSS_SUSPICIOUSNESS
CDSS_PATHOLOGICAL_GUILT--CDSS_MORNING_DEPRESSION
IL6--CDSS_OBSERVED_DEPRESSION
CDSS_OBSERVED_DEPRESSION--sex
IFNG--CDSS_GUILTY_REFERENCE
TNFA--CDSS_HOPELESSNESS
IFNG--CDSS_DEPRESSION
CDSS_SELF_DEPRECATION--age
CDSS_DEPRESSION--CDSS_GUILTY_REFERENCE
CDSS_OBSERVED_DEPRESSION--age
IL6--CDSS_GUILTY_REFERENCE
bmi_base--sex
CDSS_HOPELESSNESS--PANSSB_T
CDSS_SELF_DEPRECATION--PANSSB_T
IFNG--PANSS_SUSPICIOUSNESS
CDSS_OBSERVED_DEPRESSION--bmi_base
IL6--IFNG
age--sex
CDSS_PATHOLOGICAL_GUILT--sex
CDSS_PATHOLOGICAL_GUILT--bmi_base
CDSS_EARLY_WAKENING--bmi_base
CDSS_MORNING_DEPRESSION--age
IFNG--CDSS_MORNING_DEPRESSION
CDSS_GUILTY_REFERENCE--CDSS_SUICIDE
CDSS_HOPELESSNESS--PANSS_SUSPICIOUSNESS
IFNG--PANSSB_T
CDSS_EARLY_WAKENING--PANSSB_T
IFNG--CDSS_SUICIDE
IFNG--CDSS_PATHOLOGICAL_GUILT
IFNG--age
CDSS_MORNING_DEPRESSION--sex
CDSS_DEPRESSION--sex
CDSS_HOPELESSNESS--age
age--PANSSB_T
IFNG--sex
CDSS_GUILTY_REFERENCE--sex
IL6--CDSS_HOPELESSNESS
CDSS_SUICIDE--bmi_base
CDSS_MORNING_DEPRESSION--bmi_base
CDSS_GUILTY_REFERENCE--CDSS_EARLY_WAKENING
TNFA--CDSS_SELF_DEPRECATION
CDSS_EARLY_WAKENING--sex
PANSS_SUSPICIOUSNESS--PANSSB_T
CDSS_SELF_DEPRECATION--PANSS_SUSPICIOUSNESS
bmi_base--PANSSB_T
TNFA--PANSS_SUSPICIOUSNESS
IFNG--CDSS_OBSERVED_DEPRESSION
CDSS_DEPRESSION--bmi_base
CDSS_SUICIDE--age
TNFA--PANSSB_T
TNFA--CDSS_EARLY_WAKENING
CDSS_DEPRESSION--age
sex--PANSSB_T
−0.50
−0.25
0.00
0.25
0.50

Case-dropping bootstrapped procedures. The Figures below show the CS-coefficients for edge-weights for the network comprising treatment allocation, influential pro-inflammatory cytokines, and symptoms of the depression and suspiciousness community in the placebo group. The CS-co-efficient was 0.36.

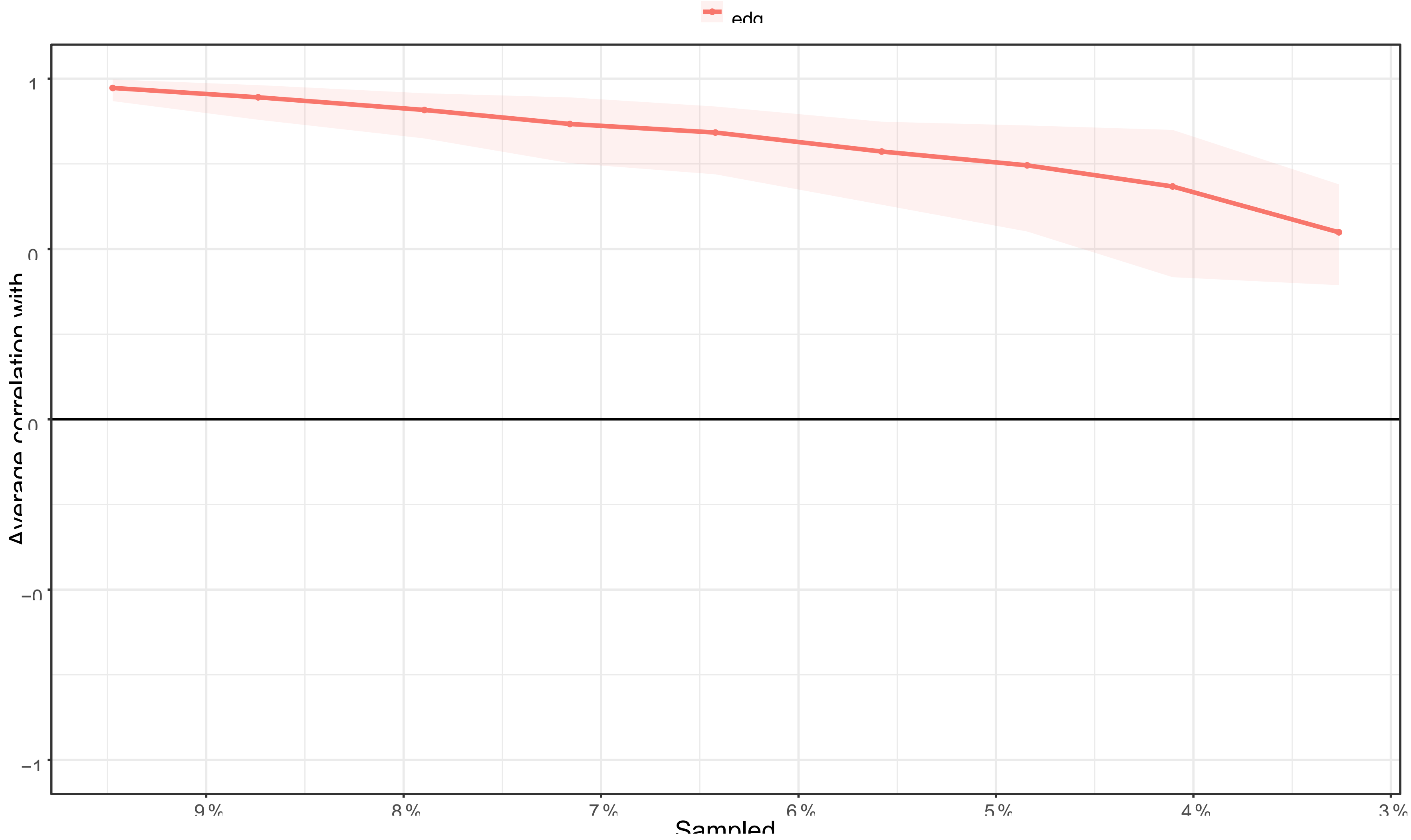

Supplementary materials for network of influential pro-inflammatory cytokines and symptoms whilst controlling for covariates at 6-month follow-up in MINOCYCLINE group

Nonparametric bootstrapped confidence intervals. The Figure below presents the nonparametric bootstrapped confidence intervals of estimated edge-weights for the network comprising influential pro-inflammatory cytokines and symptoms of the depression and suspiciousness community whilst controlling for covariates in the minocycline group. As can be seen, there were wide bootstrapped CIs around the estimated edge-weights, suggesting that relative edge strength should be interpreted with caution. The *TNF-α—IL6* association was estimated to be above zero in 49% of the 1000 nonparametric bootstrapped procedures, *TNF-α— IFN-γ* in 28%, *IL6—IFN-γ* in 41%, and *IL6—observed depression* in 56%.

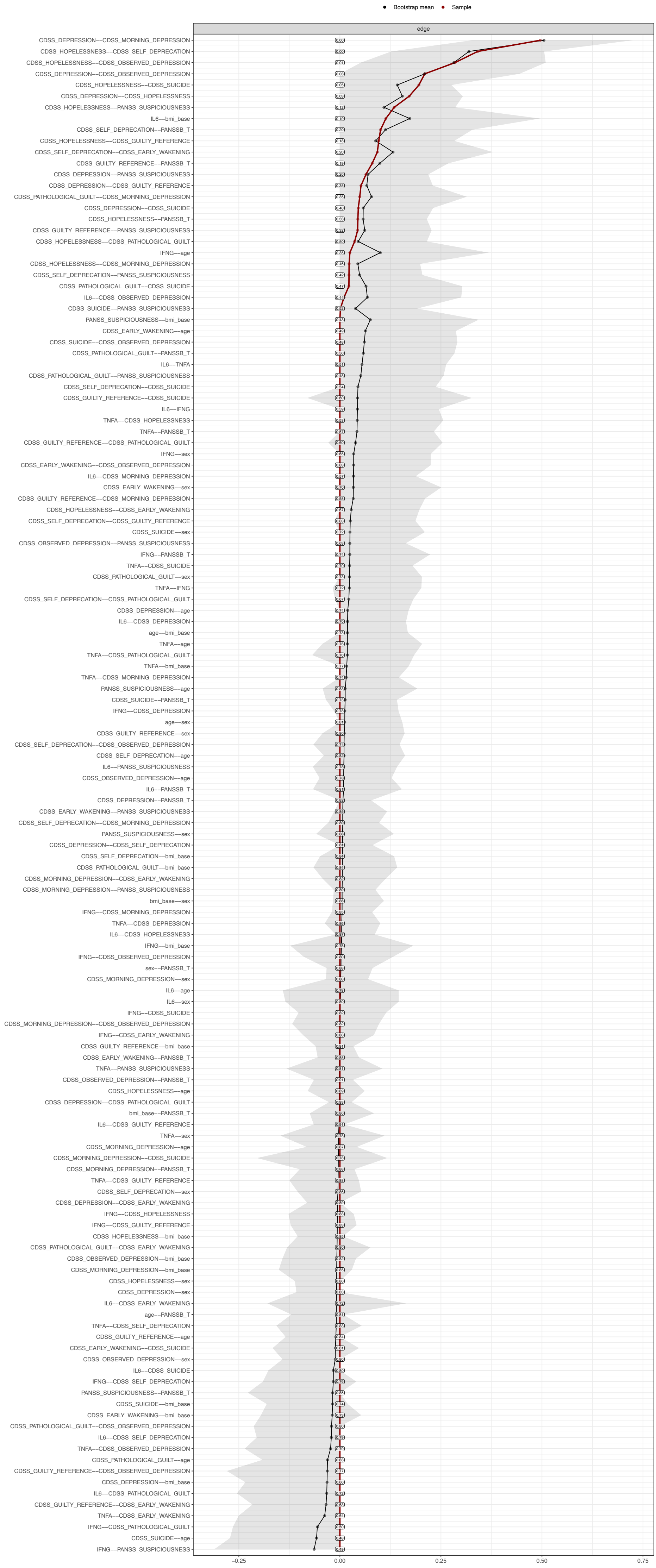
Bootstrap mean
Sample
edge
CDSS_DEPRESSION--CDSS_MORNING_DEPRESSION
CDSS_HOPELESSNESS--CDSS_SELF_DEPRECATION
CDSS_HOPELESSNESS--CDSS_OBSERVED_DEPRESSION
CDSS_DEPRESSION--CDSS_OBSERVED_DEPRESSION
CDSS_HOPELESSNESS--CDSS_SUICIDE
CDSS_DEPRESSION--CDSS_HOPELESSNESS
CDSS_HOPELESSNESS--PANSS_SUSPICIOUSNESS
IL6--bmi_base
CDSS_SELF_DEPRECATION--PANSSB_T
CDSS_HOPELESSNESS--CDSS_GUILTY_REFERENCE
CDSS_SELF_DEPRECATION--CDSS_EARLY_WAKENING
CDSS_GUILTY_REFERENCE--PANSSB_T
CDSS_DEPRESSION--PANSS_SUSPICIOUSNESS
CDSS_DEPRESSION--CDSS_GUILTY_REFERENCE
CDSS_PATHOLOGICAL_GUILT--CDSS_MORNING_DEPRESSION
CDSS_DEPRESSION--CDSS_SUICIDE
CDSS_HOPELESSNESS--PANSSB_T
CDSS_GUILTY_REFERENCE--PANSS_SUSPICIOUSNESS
CDSS_HOPELESSNESS--CDSS_PATHOLOGICAL_GUILT
IFNG--age
CDSS_HOPELESSNESS--CDSS_MORNING_DEPRESSION
CDSS_SELF_DEPRECATION--PANSS_SUSPICIOUSNESS
CDSS_PATHOLOGICAL_GUILT--CDSS_SUICIDE
IL6--CDSS_OBSERVED_DEPRESSION
CDSS_SUICIDE--PANSS_SUSPICIOUSNESS
PANSS_SUSPICIOUSNESS--bmi_base
CDSS_EARLY_WAKENING--age
CDSS_SUICIDE--CDSS_OBSERVED_DEPRESSION
CDSS_PATHOLOGICAL_GUILT--PANSSB_T
IL6--TNFA
CDSS_PATHOLOGICAL_GUILT--PANSS_SUSPICIOUSNESS
CDSS_SELF_DEPRECATION--CDSS_SUICIDE
CDSS_GUILTY_REFERENCE--CDSS_SUICIDE
IL6--IFNG
TNFA--CDSS_HOPELESSNESS
TNFA--PANSSB_T
CDSS_GUILTY_REFERENCE--CDSS_PATHOLOGICAL_GUILT
IFNG--sex
CDSS_EARLY_WAKENING--CDSS_OBSERVED_DEPRESSION
IL6--CDSS_MORNING_DEPRESSION
CDSS_EARLY_WAKENING--sex
CDSS_GUILTY_REFERENCE--CDSS_MORNING_DEPRESSION
CDSS_HOPELESSNESS--CDSS_EARLY_WAKENING
CDSS_SELF_DEPRECATION--CDSS_GUILTY_REFERENCE
CDSS_SUICIDE--sex
CDSS_OBSERVED_DEPRESSION--PANSS_SUSPICIOUSNESS
IFNG--PANSSB_T
TNFA--CDSS_SUICIDE
CDSS_PATHOLOGICAL_GUILT--sex
TNFA--IFNG
CDSS_SELF_DEPRECATION--CDSS_PATHOLOGICAL_GUILT
CDSS_DEPRESSION--age
IL6--CDSS_DEPRESSION
age--bmi_base
TNFA--age
TNFA--CDSS_PATHOLOGICAL_GUILT
TNFA--bmi_base
TNFA--CDSS_MORNING_DEPRESSION
PANSS_SUSPICIOUSNESS--age
CDSS_SUICIDE--PANSSB_T
IFNG--CDSS_DEPRESSION
age--sex
CDSS_GUILTY_REFERENCE--sex
CDSS_SELF_DEPRECATION--CDSS_OBSERVED_DEPRESSION
CDSS_SELF_DEPRECATION--age
IL6--PANSS_SUSPICIOUSNESS
CDSS_OBSERVED_DEPRESSION--age
IL6--PANSSB_T
CDSS_DEPRESSION--PANSSB_T
CDSS_EARLY_WAKENING--PANSS_SUSPICIOUSNESS
CDSS_SELF_DEPRECATION--CDSS_MORNING_DEPRESSION
PANSS_SUSPICIOUSNESS--sex
CDSS_DEPRESSION--CDSS_SELF_DEPRECATION
CDSS_SELF_DEPRECATION--bmi_base
CDSS_PATHOLOGICAL_GUILT--bmi_base
CDSS_MORNING_DEPRESSION--CDSS_EARLY_WAKENING
CDSS_MORNING_DEPRESSION--PANSS_SUSPICIOUSNESS
bmi_base--sex
IFNG--CDSS_MORNING_DEPRESSION
TNFA--CDSS_DEPRESSION
IL6--CDSS_HOPELESSNESS
IFNG--bmi_base
IFNG--CDSS_OBSERVED_DEPRESSION
sex--PANSSB_T
CDSS_MORNING_DEPRESSION--sex
IL6--age
IL6--sex
IFNG--CDSS_SUICIDE
CDSS_MORNING_DEPRESSION--CDSS_OBSERVED_DEPRESSION
IFNG--CDSS_EARLY_WAKENING
CDSS_GUILTY_REFERENCE--bmi_base
CDSS_EARLY_WAKENING--PANSSB_T
TNFA--PANSS_SUSPICIOUSNESS
CDSS_OBSERVED_DEPRESSION--PANSSB_T
CDSS_HOPELESSNESS--age
CDSS_DEPRESSION--CDSS_PATHOLOGICAL_GUILT
bmi_base--PANSSB_T
IL6--CDSS_GUILTY_REFERENCE
TNFA--sex
CDSS_MORNING_DEPRESSION--age
CDSS_MORNING_DEPRESSION--CDSS_SUICIDE
CDSS_MORNING_DEPRESSION--PANSSB_T
TNFA--CDSS_GUILTY_REFERENCE
CDSS_SELF_DEPRECATION--sex
CDSS_DEPRESSION--CDSS_EARLY_WAKENING
IFNG--CDSS_HOPELESSNESS
IFNG--CDSS_GUILTY_REFERENCE
CDSS_HOPELESSNESS--bmi_base
CDSS_PATHOLOGICAL_GUILT--CDSS_EARLY_WAKENING
CDSS_OBSERVED_DEPRESSION--bmi_base
CDSS_MORNING_DEPRESSION--bmi_base
CDSS_HOPELESSNESS--sex
CDSS_DEPRESSION--sex
IL6--CDSS_EARLY_WAKENING
age--PANSSB_T
TNFA--CDSS_SELF_DEPRECATION
CDSS_GUILTY_REFERENCE--age
CDSS_EARLY_WAKENING--CDSS_SUICIDE
CDSS_OBSERVED_DEPRESSION--sex
IL6--CDSS_SUICIDE
IFNG--CDSS_SELF_DEPRECATION
PANSS_SUSPICIOUSNESS--PANSSB_T
CDSS_SUICIDE--bmi_base
CDSS_EARLY_WAKENING--bmi_base
CDSS_PATHOLOGICAL_GUILT--CDSS_OBSERVED_DEPRESSION
IL6--CDSS_SELF_DEPRECATION
TNFA--CDSS_OBSERVED_DEPRESSION
CDSS_PATHOLOGICAL_GUILT--age
CDSS_GUILTY_REFERENCE--CDSS_OBSERVED_DEPRESSION
CDSS_DEPRESSION--bmi_base
IL6--CDSS_PATHOLOGICAL_GUILT
CDSS_GUILTY_REFERENCE--CDSS_EARLY_WAKENING
TNFA--CDSS_EARLY_WAKENING
IFNG--CDSS_PATHOLOGICAL_GUILT
CDSS_SUICIDE--age
IFNG--PANSS_SUSPICIOUSNESS
-0.25
0.00
0.25
0.50
0.75

Case-dropping bootstrapped procedures. The Figures below show the CS-coefficients for edge-weights for the network comprising treatment allocation, influential pro-inflammatory cytokines, and symptoms of the depression and suspiciousness community in the placebo group. The CS-co-efficient was 0.43.

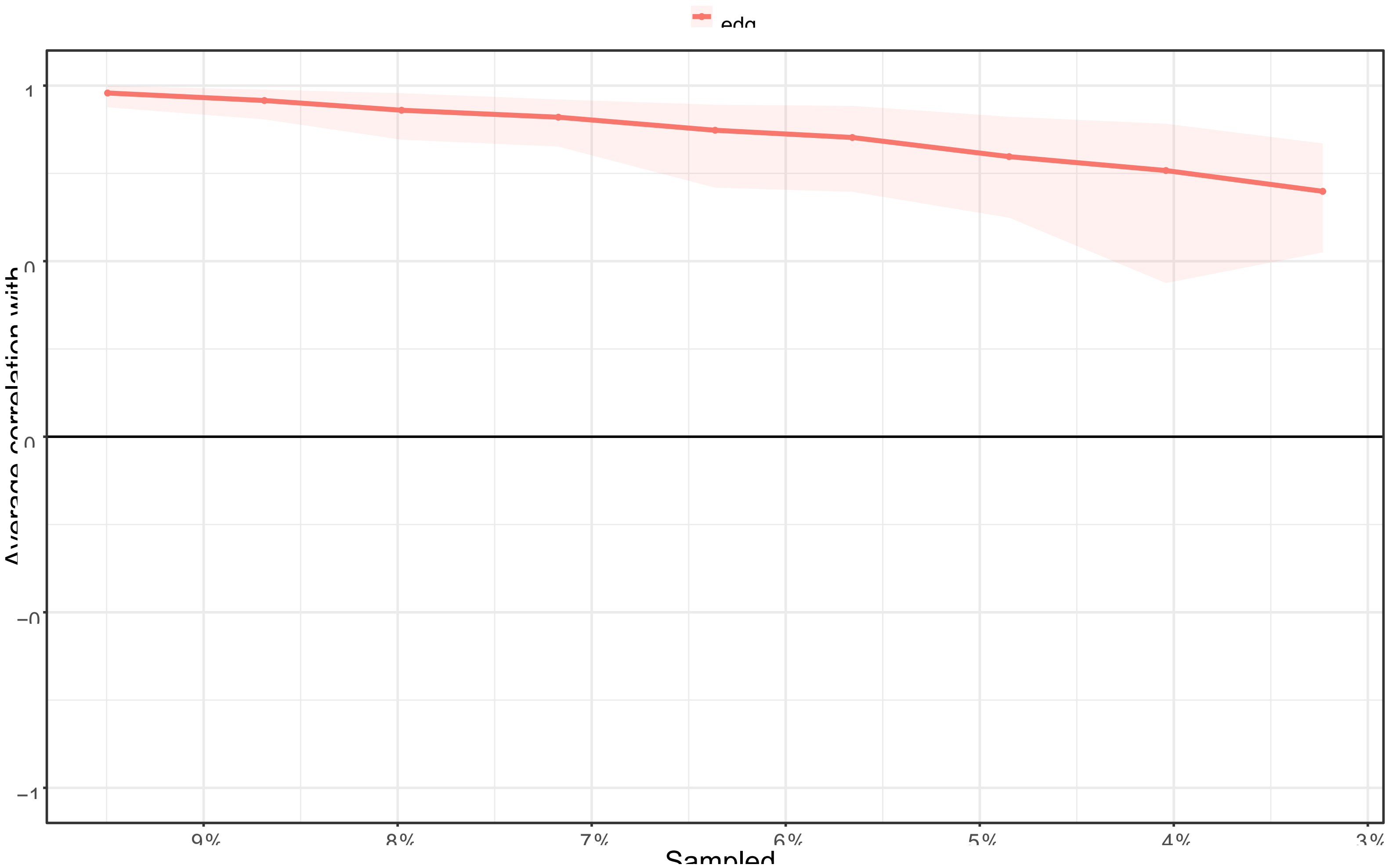

Network of influential pro-inflammatory cytokines and symptoms of the positive symptom community in placebo (A, C) and minocycline group (B, D) at six months

A B

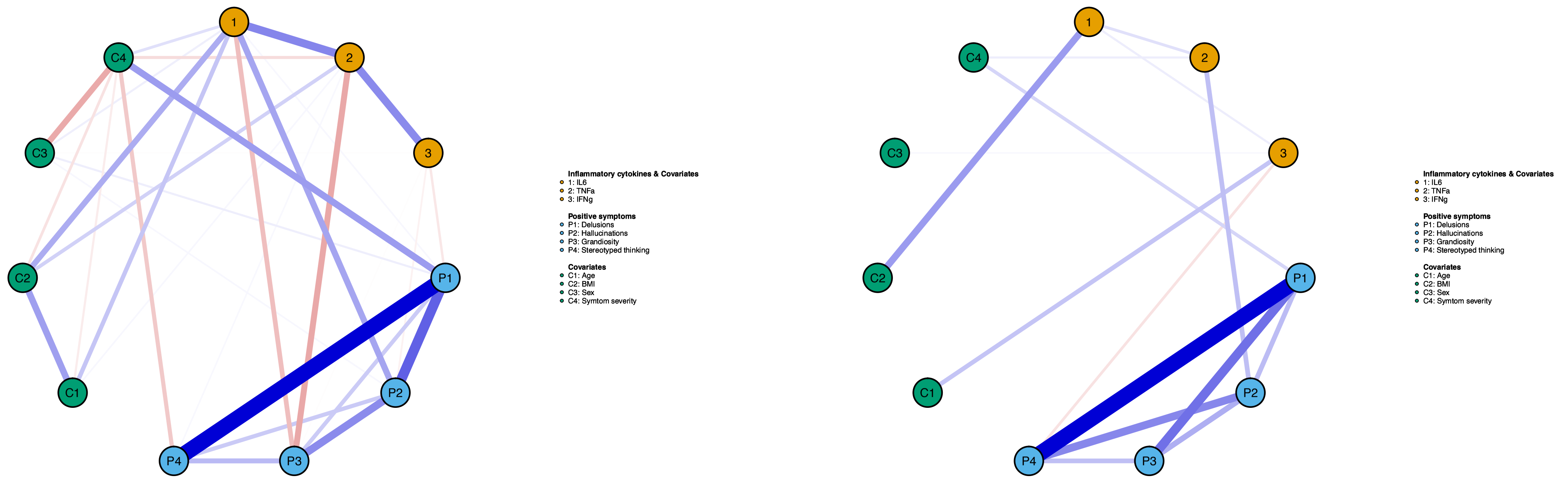
Inflammatory cytokines & Covariates
1: IL6
2: TNFa
3: IFNg
Positive symptoms
P1: Delusions
P2: Hallucinations
P3: Grandiosity
P4: Stereotyped thinking
Covariates
C1: Age
C2: BMI
C3: Sex
C4: Symtom severity

A B

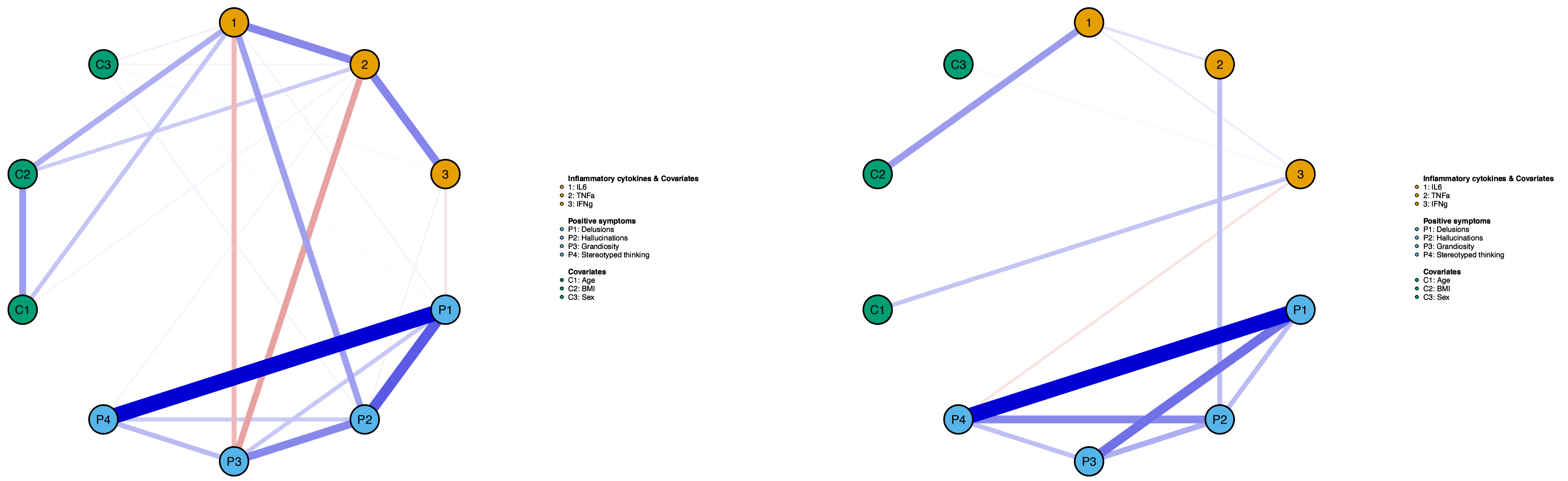
Inflammatory cytokines & Covariates
1: IL6
2: TNFa
3: IFNg
Positive symptoms
P1: Delusions
P2: Hallucinations
P3: Grandiosity
P4: Stereotyped thinking
Covariates
C1: Age
C2: BMI
C3: Sex
